# A landscape of checkpoint blockade resistance in cancer: underlying mechanisms and current strategies to overcome resistance

**DOI:** 10.1080/15384047.2024.2308097

**Published:** 2024-02-02

**Authors:** Ginette S. Santiago-Sánchez, Kellsye P. Fabian, James W. Hodge

**Affiliations:** Center for Immuno-Oncology, Center for Cancer Research, National Cancer Institute, National Institutes of Health, Bethesda, MD, USA

**Keywords:** Immune checkpoint inhibitors (ICI), immune checkpoint blockade, checkpoint blockade resistance, immunotherapy resistance, checkpoint blockade resistance mechanisms

## Abstract

The discovery of immune checkpoints and the development of immune checkpoint inhibitors (ICI) have achieved a durable response in advanced-stage cancer patients. However, there is still a high proportion of patients who do not benefit from ICI therapy due to a lack of response when first treated (primary resistance) or detection of disease progression months after objective response is observed (acquired resistance). Here, we review the current FDA-approved ICI for the treatment of certain solid malignancies, evaluate the contrasting responses to checkpoint blockade in different cancer types, explore the known mechanisms associated with checkpoint blockade resistance (CBR), and assess current strategies in the field that seek to overcome these mechanisms. In order to improve current therapies and develop new ones, the immunotherapy field still has an unmet need in identifying other molecules that act as immune checkpoints, and uncovering other mechanisms that promote CBR.

## 1. Introduction

Immunotherapy has revolutionized how advanced-stage cancers are treated by shifting the focus of the treatment from directly killing the tumor cells to activating the host’s immune system to target the tumor.^1^ One breakthrough in cancer immunotherapy has been possible due to the discovery of immune checkpoints (i.e., cytotoxic T lymphocyte antigen-4 (CTLA-4), program cell-death 1 (PD-1), and program cell-death ligand 1 (PD-L-1)).^2–5^ Under homeostasis conditions, the role of immune checkpoints, along with costimulatory signals, is to maintain self-tolerance and regulate the type and magnitude of the immune response.^2^ However, tumor cells can dysregulate the signaling of immune checkpoints to allow the evasion of tumor cells.^3^ Based on the immense role of checkpoint signaling in cancer, targeting checkpoint molecules is a rational strategy to promote anti-tumor immunity and, indeed, several immune checkpoint inhibitors (ICI) have been developed and proven effective in the clinical setting.^1,4^

CTLA-4 is an immunoglobulin-related receptor that is upregulated on conventional T cells following activation.^5,6^ As a homolog of CD28, it also binds CD80 and CD86 expressed on antigen-presenting cells (APCs) but at a higher affinity, effectively competing with CD28 for ligand binding. In contrast to CD28, CTLA-4 attenuates T cell responses potentially through various reported mechanisms such as interfering with molecules downstream of CD28 and the T cell receptor (TCR).^7,8^ In addition, CTLA-4 is highly expressed on regulatory T cells (Tregs) and is believed to be important in Treg homeostasis and function.^9^ Ipilimumab, a human monoclonal antibody (mAb) targeting the immunosuppressor molecule CTLA-4, was the first US Food and Drug Administration (FDA)-approved ICI for the treatment of metastatic, late-stage melanoma patients.^10–12^ The approval of ipilimumab was based on the results of the MDX010–020 clinical trial (NCT00094653) showing that among 676 randomized patients, 20% and 16% of the patients survived ≥2 years and ≥3 years, respectively.^4^ This was a significant improvement in overall survival (OS) over chemotherapy and cytokine-based therapies, which were used as standard-of-care (SOC) treatment for this patient population.^13^ Currently, ipililumab in combination with nivolumab is approved for colorectal cancer (CRC), hepatocellular carcinoma (HCC), mesothelioma, and non-small cell lung cancer [NSCLC].^2,14^ For melanoma, ipilimumab is approved as monotherapy or in combination with nivolumab.

The discovery of PD-1—a receptor expressed on activated T cells and its ligand PD-L1, expressed on tumor cells – identified another immunosuppressive mechanism that inactivates TCR and/or CD28 signaling, thereby impairing the normal function of cytotoxic effector T cells (Teff). Nivolumab was the first anti-PD-1 blocking antibody that was approved for treatment of inoperable or metastatic melanoma based on the CheckMate 037 (NCT01721746) clinical trial.^15^ The trial demonstrated that a greater proportion of advanced melanoma patients achieved an objective response and fewer toxic effects compared to the alternative chemotherapeutic regimen.^15^ Since then, other anti-PD-1 therapies such as pembrolizumab, cemiplimab, and dostarlimab have also been approved.^2^ Targeting PD-L1 is also a viable approach to block the PD-1/PD-L1 inhibition pathway.

The JAVELIN Merkel 200 clinical trial (NCT02155647) paved the way for the approval of the PD-L1 blocking antibody, avelumab, for the treatment of metastatic Merkel carcinoma (MCC).^12,16^ In this study, 31.8% (28 out of 88) of MCC patients whose disease had progressed on or after chemotherapy experienced objective responses with avelumab treatment.^16^ Avelumab was also later approved for patients with locally advanced or metastatic urothelial carcinoma (mUC).^12,17^ Durvalumab and atezolizumab are among the antibodies blocking PD-L1 that have been FDA-approved for the treatment of patients with locally advanced mUC.^12,18^ Collectively, ICIs targeting the PD-1/PD-L1 axis are therapeutic options for basal cell carcinoma (BCC), cutaneous squamous cell carcinoma (CSCC), NSCLC, CRC, HCC, Hodgkin lymphoma (HL), head and neck squamous cell carcinoma (HNSCC), mesothelioma, renal cell carcinoma (RCC), breast cancer (BC), large B cell lymphoma, and endometrial, esophageal, and gastric carcinomas.^2^

The blockade of multiple checkpoints as a combinatorial therapy has improved the immunomodulatory ability of the immune system in achieving tumor cell clearance. The combination of ipilimumab plus nivolumab has also been FDA-approved for the treatment of advanced unresectable/metastatic melanoma cancer patients, after showing an improvement in overall response rate (ORR), progression-free survival (PFS), and OS against the SOC chemotherapy (sunitinib).^12,15,19,20^ Furthermore, the combination has resulted in improved PFS when compared to ipilimumab alone in melanoma.^20^ In fact, except for melanoma, approved indications of ipilimumab are usually in combination with nivolumab.^2^

Despite the durable response observed with immune checkpoint blockade (ICB) as part of the SOC for the treatment of patients with a broad range of advanced-stage cancers, only a limited population of patients benefit from ICI therapy.^1,21,22^ The lack of response to ICI, also called checkpoint blockade resistance (CBR), can be categorized as primary or acquired.^1,22,23^ Primary CBR is observed in patients who do not respond to initial treatment, while acquired CBR is defined as confirmed early response to the therapy, but with eventual disease progression after prolonged treatment.^1,22,23^ For example, primary CBR is observed in metastatic BC patients,^24,25^ in about 45–70% of melanoma patients,^19,26^ and 7–27% of NSCLC patients.^27,28^

In conjunction with efforts to understand the mechanisms that result in CBR, methods to overcome CBR are being developed, such as targeting new immune checkpoints and costimulatory molecules or testing new combination therapies.^29–33^ Prevailing strategies are focused on transforming immunological “cold” tumors into “hot” tumors^30,34,35^ by increasing tumoral T cell infiltration, enhancing cytotoxic T cell function,^30,32,36^ repolarizing immunosuppressive population,^31–33,37^ or overcoming tumor-intrinsic resistance mechanisms related to loss of major histocompatibility complex class I (MHC-I), aberrations in antigen processing machinery (APM), and interferon gamma (IFN-γ) pathways.^33,38–40^ In this review, we will discuss the response of ICI in the real world and how this compares to the responses observed in clinical trials, as well as the underlying mechanisms promoting primary and acquired CBR. Finally, we aim to discuss current efforts to suppress these mechanisms that lead to CBR with the end goal of improving patient response to ICI therapy.

## 2. Contrasting responses to immune checkpoint blockade in the real-world

The response of patients treated with ICIs in the “real world” occasionally looks different from the responses observed and reported by controlled clinical trials run in different institutions. While there are data showing the consistency between the PFS and objective response rate observed in ICI-treated patients in the real world and the responses reported in the clinical trials,^41–43^ other studies have highlighted a gap between the outcomes of immunotherapy reported by clinical trials (efficacy) and the outcomes observed in real-world (effectiveness) across several cancer types.^14,44^ For example, data support the groundbreaking efficacy and durable response achieved by ICIs for the treatment of advanced-stage cancers, specifically in melanoma,^45,46^ advanced MCC,^47,48^ RCC,^49^ and NSCLC.^49^ Interestingly, these cancers have in common a high density of immune subsets infiltrated in the tumor microenvironment (TME) and the accumulation of pro-inflammatory cytokines that make them classified as “hot” tumors.^49,50^ These characteristics, among other factors that will be discussed later in the review, improve the response rate to ICI therapy in “hot” tumors.^50^ Specifically, in melanoma patients, median survival improved from 6 months when patients were treated with cytotoxic agents such as temozolomide to 6.5 years following treatment with anti-CTLA-4, anti-PD1 or a combination of both ICIs.^51^ A study of 60 patients with desmoplastic melanoma treated with antibodies blocking PD-1 or PD-L1 showed an objective response of 70% and 32% of them showed a complete response (CR).^45^ Another study of 230 metastatic melanoma patients showed a longer PFS using a combination of anti-CTLA-4 and anti-PD-1,^46^ highlighting the benefits of blocking different immune checkpoints to improve the immune response. However, it is important to mention that two-thirds of melanoma patients failed to achieve complete response after ICI therapy, hence the challenge of primary and acquired resistance faced by these patients.^51^

Blocking PD-1/PD-L1 axis with ICIs for the treatment of advanced MCC has achieved anti-tumor activity in ~ 30–60% of the patients as well as improvement in PFS and OS compared to patients receiving chemotherapy.^52–55^ Indeed, data from retrospective analyses confirmed the optimistic overview to continue using ICIs for the treatment of metastatic MCC patients.^52,56^ This study evaluated patients from two large academic medical centers who were treated with alternative ICIs after progressive disease (PD). Anti-CTLA-4 alone or in combination with anti-PD-1 revealed an overall response (OR) in 31% of the patients; one patient with MCC refractory – anti-PD-1 and anti-CTLA-4–had tumor regression with anti-PD-L1.^52^ It is worth mentioning that this study is the largest case series to date describing the anti-tumor efficacy of anti-PD-L1 as a second-line treatment for MCC patients with anti-PD-1 refractory disease.

NSCLC, as one of the most common and deadliest malignancies worldwide, also benefits from blocking the immune checkpoints PD-1, PD-L1, and CTLA-4 alone or in combination with SOC.^29^ Approximately 20–30% of NSCLC patients respond to ICI treatment and several studies continue to evaluate combination therapies to improve the response rate.^29^ Despite the progress observed, response to ICI is not always compared equally to the response reported by clinical trials. For example, a retrospective analysis of stage-IV NSCLC patients treated with pembrolizumab as a first-line treatment and nivolumab as a second-line treatment showed that the OS in real world was significantly shorter in patients receiving pembrolizumab as a first-line treatment compared to ORR as reported in clinical trials.^14^ The PFS of patients in pembrolizumab or nivolumab cohorts was comparable between real-world and trials.^14^ Likewise, another retrospective study with a cohort of 19,529 Medicare patients with NSCLC, ranging from 66 to 89 years old, showed an unadjusted median survival of 11.4 months among patients receiving single-agent pembrolizumab, which was 15 months shorter than the median survival of pembrolizumab-treated participants in the KEYNOTE-024 trial.^44,57^ In many studies, the use of chemoimmunotherapy for cancer patients with advanced stages has been incorporated into their SOC treatment regimen^58–61^; however, this study also showed that the unadjusted median survival for patients receiving chemoimmunotherapy (platinum/pemetrexed/pembrolizumab) was 12.9 months, approximately 10 months shorter than for participants in the KEYNOTE-189 trial who received the same regimen^44,62^

In total contrast to the response observed in “hot” tumors toward ICIs, the response documented in “cold” tumors is poor.^49^ “Cold” tumors such as prostate, pancreatic, and most colorectal cancers are characterized for being non-T cell infiltrated (non-inflamed) and largely resistant to ICI therapy.^49,50^ As of today, the only ICI FDA-approved treatment for prostate cancer patients is pembrolizumab (anti-PD-1), but only for metastatic castration-resistant prostate cancer (mCRPC) patients with high tumor mutational burden (TMB-H), high microsatellite instability (MSI-H), or mismatch repair deficiency (MMR-D).^63^ However, several clinical trials continue to show the limited response of mCRPC patients to single-agent ICI therapy, including pembrolizumab and other ICIs such as atezolizumab (anti-PD-L1) and ipilimumab (anti-CTLA-4).^63^ Besides poor infiltration in “cold tumors,” these tumors rarely express PD-L1 (immunologically ignorant) and show low expression of neoantigens and immunosuppressive TME, all contributing factors to their unresponsiveness to checkpoint blockade.^49^

## 3. Checkpoint blockade resistance

CBR needs to be discussed not only in terms of the biological concepts, but also in the complex scenario of the clinical setting, making it challenging to convey a uniform clinical definition of resistance to ICB for advanced diseases.^23^ To bring consensus to defining CBR, several principles and guidelines have to be considered and established, including the underlying mechanisms driving primary or acquired resistance,^64–66^ treatment duration criteria to determine the cutoff to distinguish primary resistance,^23,66^ and treatment discontinuation criteria to determine if acquired resistance is considered even after cessation of the ICI.^23,66^ In response to this heterogenous scenario, the Society for Immunotherapy of Cancer (SITC) taskforce agreed to take into consideration the duration of drug exposure, scan requirements, and response evaluation to define both primary and acquired resistance.

First, the taskforce stated that it was critical to define a minimal exposure timeframe for patients treated with an FDA-approved PD-1 or PD-L1 inhibitor to derive any possible clinical benefit.^23^ The taskforce established a required exposure to ICI therapy of at least 6 weeks but not more than 6 months. For patients with indolent tumor types, however, this timeframe needs to be redefined.^23,67^ To determine whether a treated patient is showing immune checkpoint primary resistance, a confirmatory scan needs to be performed to validate PD.^23^ On the other hand, the taskforce disagreed on which response evaluation criteria – Response Evaluation Criteria in Solid Tumors^68^ or RECIST 1.1—was to be employed and agreed that immune-based Response Evaluation Criteria in Solid Tumors (iRECIST) could not be used as a sole criterion to determine ICI efficacy. Although iRECIST was created to address cases of mixed responses and pseudoprogression, the taskforce stated that further validation is needed.^23,68^ However, the group agreed that the use of a fluorodeoxyglucose (FDG)/positron emission tomography (PET) scan combined with metabolically active immune infiltrates may be used as an indicator of response to ICI therapy.^23^

The taskforce defined acquired resistance in advanced disease settings, otherwise known as secondary resistance, when a patient is treated for 6 months or longer, has a CR, partial response (PR), or prolonged stable disease (SD) – confirmed by scan – for more than 6 months and then presents PD in the setting of ongoing treatment.^23^ Members of the taskforce did not reach a consensus regarding the requirement of confirmatory imaging for validating secondary resistance. Two of the three groups recommended a scan within 4–12 weeks after evidence of PD, including verification of ≥2 metastatic sites/lesions for patients with multiple metastases, for acquired resistance confirmation.^23^

Uncommon response patterns represent an additional challenge in defining clinical resistance to ICIs.^23,69^ One of these patterns is known as pseudoprogression and describes patients who appear to have PD after radiographic confirmation, but experienced tumor shrinkage months after treatment cessation.^23,69,70^ This type of response was first observed in a phase II clinical trial that evaluated the efficacy of ipilimumab in metastatic melanoma patients.^71^ In this study, Wolchok et al. showed that 9.7% (22 out of 227) of the treated patients were characterized with PD – following World Health Organization (WHO) and iRECIST guidelines – in spite of having clinical responses (PR and SD).^70,71^ Clinical trial protocols did not require patient follow-up after PD was observed, thus limiting data collection for those showing a response after leaving the clinical trial. Accordingly, the number of patients with pseudoprogression may have been underestimated.^71^ Current data support that pseudoprogression occurs in approximately 5–10% of the patients receiving anti-CTLA-4 or anti-PD-L1 therapy, across several solid tumor types.^23,70,72^ Building on these findings, a novel benchmark designated as immune-related response criteria (irRC) is currently used to differentiate pseudoprogression from true progression and may provide a more comprehensive evaluation of the response toward the immuno-oncology (IO) agent.^71,72^ As such, a study evaluating irRC and RECIST criteria in advanced melanoma patients treated with pembrolizumab showed that the sole use of RECIST criteria may have underestimated the benefit of the checkpoint in approximately 15% of the patients.^73^

## 4. Underlying mechanism associated to ICI resistance

### 4.1 T cells exclusion and dysfunction in the TME

The density of preexisting T cells in the TME as well as their functionality are two of the factors used to determine the outcome of ICI therapy.^1^ Immune-excluded tumors – “cold tumors” – lack infiltrating effector T cells and are less prone to respond to ICI therapy. This lack of response may be due to primary resistance conferred by mutations and dysregulation of cell signaling pathways in tumor cells, resulting in poor T cell recruitment and T cell dysfunction.^1,39^ For example, a study in highly aggressive HCC determined that low T cell numbers may be associated with deletions in the MAX/TP53 genes, which in turn resulted in the downregulation of TP53-related chemokines that are pivotal in T cell recruitment.^74,75^

**Figure 1.**
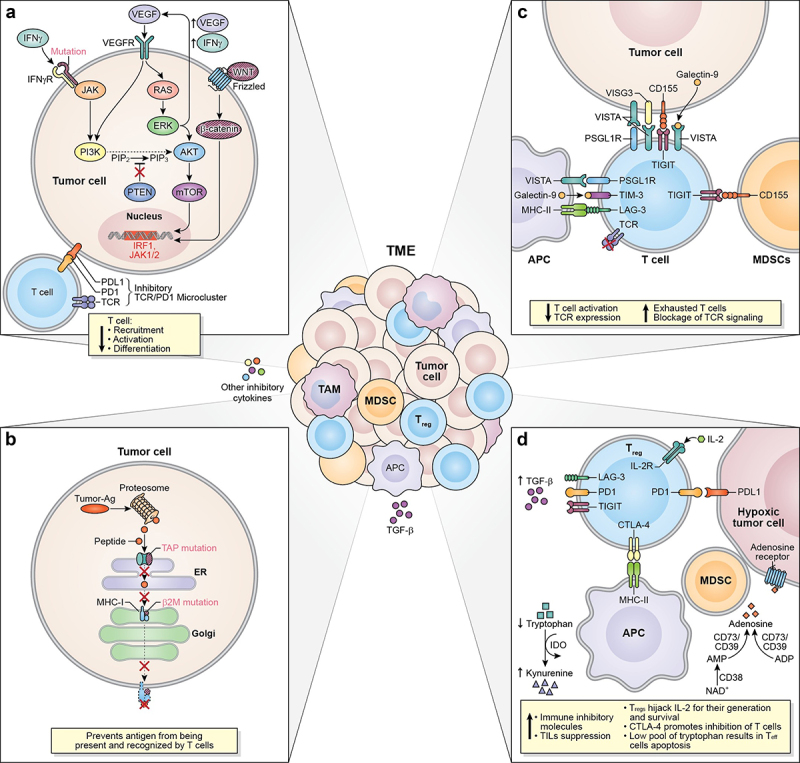
Mechanism associated to ICI resistance. The TME represents a complex interaction between tumor cells, immune cells, and inhibitory cytokines leading to a plethora of mechanisms associated to ICI resistance. (a) Defects in T cells effector function and their exclusion from the TME can occur as a result of dysregulation of MAPK/ERK cell signaling pathways in tumor cells inducing the production of VEGF and IL-8, having a net inhibitory effect on the recruitment of T cells and defects in T-cell activation and differentiation. Due to the crosstalk between the MAPK and the PI3K signaling pathways, an oncogenic mutation in MAPK pathway or a loss of PTEN expression can cause the enhancement of PI3K-AKT signaling as well. Similarly, a mutation or silencing of β-catenin or tumor suppressor wnt protein, results in dysregulation of the WNT/β-catenin pathway, promoting aberrant signaling of b -catenin contributing to the absence of T cell expression signature. Mutations in the IFN-g receptor and defects on the IRF1 or JAK1/2 genes can contribute to T cell desensitization and consequently promote acquired resistance to ICIs. Specifically, mutations of PD-L1, PD-L2, and JAK2 genes resulting from the amplification of the locus that contains these genes promote the formation of the PD-1/TCR inhibitory microcluster, which results in the inhibition of T cell activation. (b) Another resistance mechanism is developed after defects in the APM specifically due to mutations of B2M and TAP proteins. These mutations make the antigen unable to reach the tumor cell’s surface and be recognized and cleared out by CD8^+^ T cells. (c) the interaction between T cells, tumor cells, APCs, and immunosuppressive cells (MDSCs) throughout immune checkpoints such as VISTA, LAG-3, TIGIT, and TIM-3, triggers and inhibitory signal causing the exhaustion of T cells, blockage of TCR signaling, and decreasing T cell activation and TCR expression. (d) Immunosuppressive signaling also contributes to primary and/or acquired resistance in cancer. Tregs can restrain the effector function of immunocompetent cells by inducing checkpoint-mediated suppression (CTLA-4, PD-1, TIGIT, TIM-3, and LAG-3), competing for IL-2 binding, or by producing anti-inflammatory cytokines. As such, TGF-b is a pleiotropic cytokine involved in tumor evasion and immunotherapy resistance. The upregulation of the metabolic modulator IDO within the TME allows the depletion of tryptophan, resulting in the decrease of T cells. Another metabolic modulator associated with T cell function suppression and ICI resistance is adenosine. Several ectonucleotidases (CD39 and CD73) catalyze the conversion of ADP or AMP to adenosine or NAD^+^ to AMP (CD38) causing the upregulation of adenosine in the tumor milieu. TME: tumor microenvironment; MAPK/ERK: mitogen-activated protein kinase/extracellular signal-regulated kinase; VEGF: vascular endothelial growth factor; IL-8: interleukin-8; PTEN: phosphatase and tensin homolog deleted on chromosome 10; PI3K-AKT: phosphoinositide-3-kinase – protein kinase B/Akt; WNT/β-catenin: wingless-related integration site/ b-catenin; IRF1: interferon regulatory factor 1; JAK1/2: Janus kinase 1/2; PD-L1/L2: program cell-death ligand 1/ligand 2; PD-1/TCR inhibitory microcluster: program cell-death 1/T cell receptor inhibitory microcluster; APM: antigen presenting machinery; β2 M; β2-microglobulin; TAP:transporter associated with antigen presentation; APC: antigen presenting cells; MDSCs: myeloid-derived suppressor cells; VISTA: V-domain ig suppressor of T cell activation; LAG-3: lymphocyte activation gene 3; TIGIT: T cell ImmunoGlobulin and ImmunoTyrosine inhibitory motif (ITIM) domain; TIM-3: T cell immunoglobulin and mucin-3; IDO: indoleamine 2,3-dioxygenase 1; CTLA-4:cytotoxic T lymphocyte antigen-4; TGF-β: transforming growth factor beta ; ADP: adenosine triphosphate; AMP: adenosine monophosphate; NAD^+^: nicotinamide adenine dinucleotide.

Multiple oncogenic signaling pathways affecting primary resistance have been identified, including mitogen-activated protein kinase (MAPK)/extracellular signal-regulated kinase^76^ signaling pathways and/or loss of phosphatase and tensin homolog deleted on chromosome 10 (PTEN) expression.^1,74,77^ Dysregulated MAPK/ERK pathways induce the production of vascular endothelial growth factor (VEGF) and interleukin-8 (IL-8), resulting in an inhibitory effect on the recruitment of T cells and defects in T-cell activation and differentiation (Figure 1a).^74,78^ Different cancer types harbor somatic mutations related to the MAPK pathways, many of which had been demonstrated to be oncogenic.^79^ For example, approximately 18% of HNSCC patients harbor MAPK pathway mutations and half of these mutations are oncogenic in nature.^79,80^ As a result of the crosstalk between the MAPK and the phosphoinositide-3-kinase – protein kinase B/Akt (PI3K-AKT) signaling pathways, an oncogenic mutation in MAPK pathway – or loss of PTEN expression – will cause the enhancement of PI3K-AKT signaling, associated with resistance to checkpoint blockade resistance (Figure 1a).^1,77,81^ A study of melanoma cancer patients correlated the constitutive activation of PI3K-AKT signaling, due to PTEN loss, with low numbers and impaired function of the tumor-infiltrated lymphocytes (TILs), as well as poor outcomes after PD-1 inhibitor treatment.^81^

Another common mutation in cancer occurs in the WNT/β-catenin signaling pathway, particularly due to mutation or silencing of the Wnt protein, which acts as a tumor suppressor (Figure 1a).^82^ In melanoma cancer patients, dysregulation of the WNT/β-catenin pathway has been correlated with unresponsiveness to ICI therapy.^82^ This was corroborated in a murine melanoma model wherein continuous β-catenin signaling in the TME contributed to the absence of T cell expression signature and, consequently, resistance to anti-PD-L1 and anti-CTLA-4 therapy.^82^ The growing evidence of the relationship between the activation of WNT/β-catenin signaling and ICI resistance has given the rationale to target β-catenin pathway to enhance the efficacy of ICIs.^83^

Cancer-associated resistance also arises as a result of mutations in the IFN-γ signaling pathway (Figure 1a).^1,77^ IFN-γ is a pleiotropic cytokine with antitumor and immunomodulatory functions; thus, it plays an important role in both innate and adaptive immune responses.^84^ IFN-γ activates its receptor (IFNG1/2) which is intracellularly associated with kinases from the Janus kinase (JAK) family (JAK1 and JAK2), inducing the expression of genes involved in cell cycle regulation, apoptosis, growth inhibition, and tumor suppression.^84^ The growing data support the idea that defects in the IFN-γ signaling pathway cause T cell desensitization, allowing acquired resistance to checkpoint blockade.^1,77,85,86^ Recent data showed that biopsies from melanoma patients who did not respond to anti-CTLA-4 therapy have mutations in IFNG1/2, JAK2, and interferon regulatory factor 1 (IRF1) genes.^85^ In a separate study, two out of four metastatic melanoma patients who had progressive disease after initial objective tumor regression with pembrolizumab had loss-of-function mutations in JAK1 or JAK2 genes.^86^ Mutations in the IFN-γ signaling pathway can also foster primary or acquired resistance via constitutive PD-L1 expression or the loss of PD-L1 expression in cancer cells.^1,77^ The mutations in the first scenario arise after the amplification of the locus in chromosome 9 that contains the genes for PD-L1, PD-L2, and JAK2.^1^ The interaction of the PD-1 receptor with its ligand, PD-L1, now overexpressed in tumor cells due to the mutation, forms the PD-1/TCR inhibitory microcluster, resulting in the inhibition of T cell activation (Figure 1a).^1^ In the second scenario, in which PD-L1 expression is no longer inducible by IFN-γ, anti-PD-1/PD-L1 antibodies would not be effective, and patients would manifest primary resistance to ICI.^1,87^

### 4.2 Defects in antigen processing machinery and lack of tumor-associated antigens

The antigen processing and presentation pathway are critical in the immunosurveillance of cancer.^88,89^ Tumor antigen recognition by CD8^+^ T cells requires that for an effector CD8^+^ T cell to recognize an antigen from a tumor cell, the antigen must be expressed in the surface of the tumor cells and the T cell must be able to recognize that antigen presented by an MHC class I molecule (MHCI).^89^ In humans, MHCI molecules are heterodimers composed of heavy chains encoded by Human Leukocyte Antigen (HLA) genes (*HLA-A, HLA-B*, and *HLA-C*) and a light chain b_2_- microglobulin (B2M).^88,89^ Antigen processing is a multi-step procedure that includes antigen fragmentation by the proteosome, relocation of the antigenic peptide to the endoplasmic reticulum (ER) by specific transporter proteins, docking of the peptide on an MHC-I molecule, and the transport of the MHC-I-peptide complex to the cell surface.^89^

Evidence indicates that mutations and epigenetic modifications in the cancer DNA that alter the antigen processing and presentation machinery can contribute to checkpoint immunotherapy resistance.^1,88^ Specifically, tumor cells can develop acquired resistance through loss of surface expression of MHC-I molecules via mutations in the B2M light chain (Figure 1b).^1,88^ Without B2M, the MHC-I molecules cannot be folded and transported to the surface of the cells in order to present the antigen; thus, CD8^+^ T cells will not be able to recognize the antigen and clear out the tumor cell.^88^ In addition, mutations in genes encoding transporter proteins, such as transporters associated with antigen presentation 1/2 (TAP) 1/2, which are involved in the process of importing antigen peptides into the endoplasmic reticulum, inhibit the formation of MHC-I-peptide complexes, thereby preventing the antigen processing and presentation machinery to proceed (Figure 1b).^88^

Logically, the presence of targetable tumor antigens is a critical factor contributing to the ability of T cells to mount an immune response and to the ability to gain clinical benefits with ICI therapy.^89^ These tumor antigens can be generated through genetic diversity, missense, and silent mutations, insertions, deletions, as well as copy number gains and losses.^89^ For example, cancers with MMR-D as a result of loss of function of certain genes (*MLH-1, PMS-2, MSH-2*, and *MSH-6*) involved in the DNA repair pathway and those that have MSI-H are examples of cancers with TMB-H.^40^ Both MSI-H and MMR-D induce frameshift mutations allowing for neoantigen formation.^38,40^

The number of all the somatic mutations per megabase (Mb) in the genome of a tumor cell, also known as TMB, seems to be the driver generating the immunogenic neoantigens presented by the MHC complex.^89,90^ Cancers with a high TMB, which is broadly defined as cancers with ≤20 mutations per Mb, have a higher objective response rate to ICI therapy.^78,91–93^ As such, melanoma and NSCLC are among the malignancies with TMB-H and higher OR to anti-PD-1 therapy.^38,94^ Nonetheless, only 45% of the patients with TMB-H responded to ICI therapy, indicating the existence of other mechanisms promoting primary resistance in these cases.^91,95^ Whereas high ORR and OS are associated with tumors with TMB-H, cancers categorized as low TMB (TMB-L), defined as ≤5 mutations per Mb,^38,91^ such as pancreatic cancer and triple-negative breast cancer (TNBC), have a low probability of response to ICIs and manifest as primary or acquired resistance.^38,91,96,97^ These observations were highlighted in a study that analyzed patients across several solid tumors, excluding NSCLC and melanoma, who had TMB assessment and received ICI therapy. The study revealed that only a low percentage of patients (9%) harboring TMB-L tumors had CR/PR; conversely, 47% of the patients with TMB-H had CR/PR.^95^

### 4.3 Presence of alternative inhibitory immune checkpoints

V-domain Ig suppressor of T cell activation (VISTA), T cell immunoglobulin and mucin-3 (TIM-3), lymphocyte activation gene 3 (LAG-3), and T cell ImmunoGlobulin and ImmunoTyrosine inhibitory motif (ITIM) domain (TIGIT) are examples of alternative checkpoint molecules that elicit immune inhibitory signals that may contribute to primary and acquired resistance (Figure 1c).^1,98–100^ VISTA is a negative regulator of T cell activation and is expressed by neutrophils, monocytes, macrophages, dendritic cells, TILs, and by some human cancer cells (ovarian cancer, endometrial cancer, and CRC).^98,100–102^ Although the specific mechanisms through which VISTA exerts immune inhibition are still unclear, its role in acquired resistance is being elucidated. A study of metastatic melanoma patients treated with ipilimumab found that the majority of patients (12/18) had biopsies with significantly higher densities of VISTA^+^ lymphocytes during disease progression compared to pre-treatment.^98,103^ Interestingly, another study that analyzed tumor tissue biopsies from prostate cancer patients found that suppressive macrophages express either VISTA (26.5%) or PD-L1 (29.4%), but rarely both markers (2%).^85,98^ The presence of distinct macrophages suggests that these subsets may compensate for each other during ICI therapy.^85,98^ Overall, there is evidence that VISTA may be a compensatory inhibitory pathway that results in acquired resistance to currently available ICI therapy.^103^

LAG-3 is expressed on activated T cells, natural killer (NK) cells, B cells, and dendritic cells (DCs), and currently, the only known ligand for LAG-3 is the MHC-II molecule.^100^ LAG-3/MHC-II high-affinity interaction blocks proper TCR signaling, resulting in hampered T cell functions.^104^ Data showed that MHC-II expression in tumor cells is associated with increased response to anti-PD-1 therapy.^105^ A follow-up study demonstrated that MHC-II expression in tumors is associated with an increased infiltration of LAG-3+ TILS.^100,106^ Notably, LAG-3 expression was higher in specimens from patients who initially responded to PD-1 therapy but eventually progressed. Furthermore, LAG-3 has been observed to be co-expressed with PD-1 in exhausted or dysfunctional T cells in human tumors like ovarian cancer and melanoma.^100,107^ Additionally, LAG-3 signaling can promote Treg populations through the release of immunosuppressive cytokines like transforming growth factor beta (TGF-β) and IL-10.^104^ Collectively, the growing body of data suggests that LAG-3 upregulation is associated with primary and acquired resistance in different cancer conditions.^104,108^

The immune checkpoint molecule TIM-3 is expressed in activated human T cells, NK cells, and monocytes.^100^ High TIM-3 expression in several syngeneic models has been associated with acquired resistance toward PD-1 blockade.^109–111^ For example, Koyama et al. correlated acquired resistance after anti-PD-1 therapy with TIM-3 upregulation in a lung adenocarcinoma murine model.^110^ The same TIM-3 upregulation was observed in lung cancer patients who had PD following PD-1 blockade. Furthermore, TIM-3 expression was higher in the relapsed NSCLC patients when compared to those who were not treated with ICIs.^110^ Additionally, clinical data support that TIM-3 is universally co-expressed with PD-1 in TILs, enabling a more exhausted T cell phenotype.^100,112^ Indeed, a study associated the co-expression of PD-1 and TIM-3 with acquired resistance to anti-PD-1 therapy in NSCLC patients.^109^

TIGIT expression is restricted to CTLs, Th, Tregs, and NK cells.^113,114^ TIGIT’s inhibitory signaling occurs upon interaction with its main ligand CD155 (PVR), expressed on tumor-infiltrating myeloid cells and tumor cells.^114^ Since TIGIT binds to CD155 with a higher affinity than its competitive ligand CD226, TIGIT/CD155 interaction disrupts CD226 homodimerization, inhibiting CD226-mediated T cell activation.^113,114^ The TIGIT/CD155 complex can also reduce TCR-expression and TCR signaling.^114^ A study of melanoma correlated high CD155 and TIGIT expression with PD-1 and/or CTLA-4 primary and acquired resistance in patients with non-inflamed tumors and inflamed tumors with TMB-H.^115^ In addition, a study using a preclinical model of pancreatic adenocarcinoma (PDAC) supported the hypothesis that TIGIT blockade may overcome preexisting or acquired resistance to CD40a/PD-1 therapy.^116^

### 4.4 Immunosuppressive signaling

Tumor-cell extrinsic factors such as immunosuppressive cells, including Tregs and tumor-associated macrophages (TAMs), and inhibitory cytokines, such as TGF-β, also contribute to primary and/or acquired resistance in cancer (Figure 1d).^1,3^ In physiological conditions, the main function of Tregs is to maintain immune homeostasis; however, cancer exploits Treg cellular mechanisms to restrain the effector function of immunocompetent cells.^117^ Tregs can induce CTLA-4-mediated suppression of APCs, compete for IL-2 binding, produce anti-inflammatory cytokines, and express immune inhibitory molecules such as TIGIT, PD-1, TIM-3, and LAG-3.^117^ Indeed, a study in NSCLC, gastric cancer and melanoma have suggested the involvement of Tregs in resistance to anti-PD-1 immunotherapy.^117^ This study demonstrated that the balance between PD-1^+^CD8^+^ T cells and PD-1^+^ Tregs in the TME can better predict the outcome of PD-1 therapy than PD-L1 tumor tissue expression or TMB.^117^

TGF-β is a pleiotropic cytokine that plays a key role in maintaining immune tolerance, yet is also involved in tumor evasion and immunotherapy resistance.^118,119^ A plethora of studies on solid tumors have correlated TGF-β signaling in the TME with different mechanisms underlying ICI resistance.^118,119^ For example, TGF-β can suppress TILs and at the same time induce high expression of PD-1 and PD-L1 in T cells and tumor cells, respectively.^118^ Additionally, a clinical study showed that high TGF-β gene signature can predict the failure of ICIs in gynecological cancer patients.^119^ The role of TGF-β in promoting primary resistance is yet to be understood; however, inhibition of TGF-β in a murine model refractory to anti-PD-1 improved antitumor response and survival benefits.^118,120,121^

Metabolic modulators such as indoleamine 2,3-dioxygenase 1 (IDO) and adenosine can also play a role in fostering immunosuppressive conditions in the TME that result in immunotherapy resistance (Figure 1d).^104,122,123^ IDO is an intracellular enzyme that catalyzes the reaction that converts tryptophan into kynurenine under normal physiological conditions. It is expressed only in select tissues (mucosal tissues, placenta, eye, and pancreas) and by a small population of immune cells (DCs and eosinophils).^29,122^ However, data confirmed IDO upregulation in CRC, breast cancer, prostate cancer, and esophageal cancer.^29^ As such, expanding preclinical and clinical data correlated IDO upregulation with suppression of T cell function and ICI resistance.^29,122,124^

Adenosine accumulates in the TME through the conversion of hydrolyzed forms of adenosine triphosphate (ATP) to adenosine diphosphate (ADP) or adenosine monophosphate (AMP), and then into adenosine by ectonucleotidases (CD39 and CD73), which are overexpressed in different cancer types.^123^ High levels of adenosine in the TME may also be due to mutations or hypoxic conditions that decrease the reduction of this metabolite (Figure 1d).^123^ Another source of adenosine in the TME comes from nicotinamide adenine dinucleotide (NAD^+^) converted to AMP by CD38, a molecule that is upregulated by tumor cells and identified as another mechanism of resistance to PD-1 and PD-L1 checkpoint blockade (Figure 1d).^123^

## 5. Strategies to overcome associated mechanisms contributing to immunotherapy resistance

As described above, response to ICI is contingent on the presence, abundance, and activity of tumor-reactive T cells. Treatments to induce effector T cell activation, infiltration, and function, therefore, are strategies that can be combined with ICI to enhance antitumor response or used after ICI therapy failure (Table 1).

**Table 1.** Clinical trials recruiting patients with primary refractory or acquired secondary resistance to prior immune checkpoint treatments.

Study ID	Malignancies	Phases	Combinatorial approach
NCT04577807	Melanoma	Phase II	Lerapolturev (formerly known as PVSRIPO) (live attenuated Sabin type 1 polio vaccine, targeting CD155) 125 anti-PD-1
NCT04239040	Neuroblastoma	Phase I	GVAX vaccine (irradiated GM-CSF secreting, autologous neuroblastoma cell vaccine) Nivolumab Ipilimumab
NCT03388632	Metastatic solid tumors	Phase I	IL-15 Nivolumab Ipilimumab
NCT05533697	Advanced solid tumors	Phase I/II	mRNA-4359 vaccine (encoding for concatemerized PD-L1 and indoleamine 2,3-dioxygenase 1 (IDO1) antigens 126 Pembrolizumab
NCT03474497	Metastatic NSCLC, melanoma, RCC, or HNSCC	Phase I/II	IL-2 Pembrolizumab Radiotherapy
NCT03739931	Solid tumors or lymphoma	Phase I	mRNA-2752 (a lipid nanoparticle encapsulating mRNAs encoding human OX40L, IL-23, and IL-36γ)
NCT05764395	Unresectable metastatic melanoma	Phase II	Rigosertib (ON01910) (in vitro inhibitor of PLK1) 127 Pembrolizumab
NCT05200143	Cutaneous melanoma	Phase II	Ipilimumab Nivolumab Cabozantinib
NCT05431270	Solid tumors	Phase I	PT199 (an anti-CD73 mAb) Anti-PD-1
NCT04493203	Advanced melanoma	Phase II	Nivolumab Axitinib
NCT05723055	Classical Hodgkin lymphoma	Phase II	Nivolumab Axatilimab
NCT03333616	Advanced Rare Genitourinary Tumors	Phase II	Nivolumab Ipilimumab
NCT04862455	Recurrent/metastatic HNSCC	Phase II	NBTXR3 (hafnium oxide-containing nanoparticles) Pembrolizumab
NCT03747484	Metastatic MCC	Phase I/II	FH-MCVA2TCR (gene-modified autologous MCPyV-specific HLA-A02-restricted TCR-transduced CD4+ and CD8+ T-cells) Pembrolizumab Interferon--1b
NCT03228667	NSCLC, SCLC, UC, HNSCC, MCC, melanoma, RCC, gastric cancer, cervical cancer, HC, MSI/MMR-D CRC	Phase II	*N*-803 Pembrolizumab Nivolumab Atezolizumab
NCT04879368	AGOC	Phase III	Regorafenib Nivolumab Docetaxel
NCT03161431	Melanoma	Phase I	Pembrolizumab SX-682 (CXCR1/CXCR2 inhibitor)

NSCLC: non-small cell lung carcinoma, RCC: renal cell carcinoma, HNSCC: head and neck squamous cell carcinoma, SCLC: small cell lung carcinoma, MCC: Merkel cell carcinoma, PLK1: polo-like kinase 1, UC: urothelial carcinoma, HC: hepatocellular carcinoma, MSI: microsatellite instability, MMR-D: mismatch repair deficiency, AGOC: advanced gastro-esophageal carcinoma.

### 5.1 Overcoming deficiencies in T cell priming

**Figure 2.**
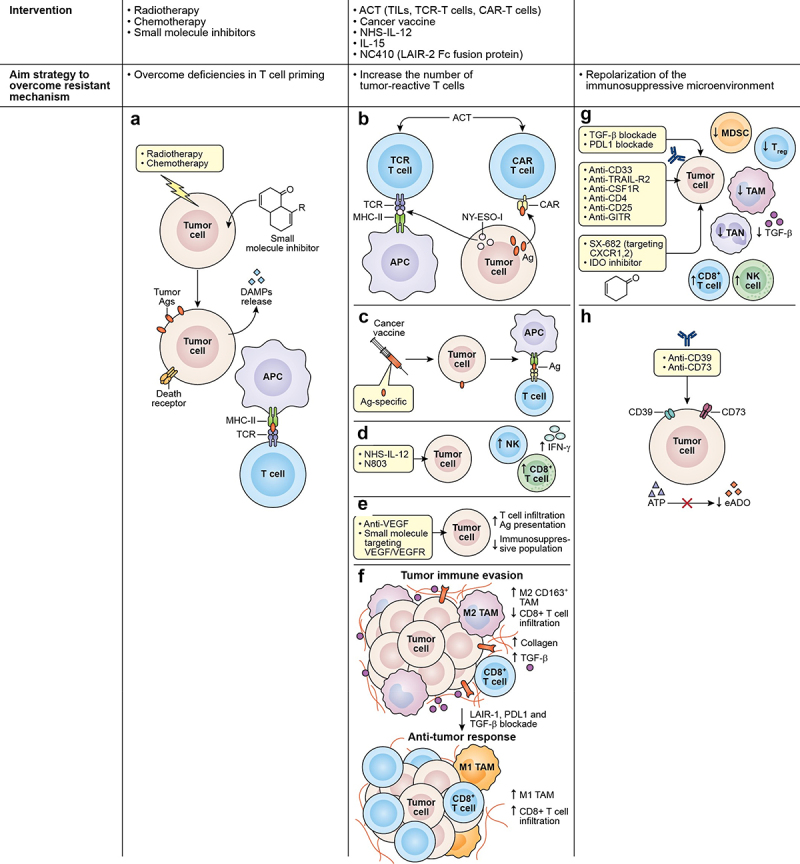
Strategies to overcome associated mechanisms contributing to immunotherapy resistance. (a) Chemotherapy, radiotherapy, and small molecule inhibitors are among the strategies used to overcome deficiencies in T-cell priming. These agents can induce ICD in tumor cells causing the release of antigens and DAMPs, allowing the recruitment and maturation of APCs and the presentation of targetable antigens to effector T cells. Other interventions are used to increase the number of tumor-reactive T cells interacting in the TME, such as (b) ACT using TCR T cell or CAR T cell. TCR T directed against specific cancer antigens (testis antigen, NY-ESO-1) can recognize the antigen through MCH molecules and CAR T cells act in an MHC-independent manner targeting cell surface antigens. (c) the use of cancer vaccines is also used to expand tumor-specific T cell populations, broaden the T cell repertoire, and promote the transport of T cells to tumor lesions. Additionally, the use of (d) immunostimulatory cytokines such as NHS-IL-12 or N803 can promote the enhancement of effector cell recruitment and boost CD8^+^ T cell and NK cell cytolytic functions. (e) physical barriers also prevent the infiltration of effector T cells in the TME, and tumor vasculature represents one of these barriers. Therefore, VEGF targeting using small molecule inhibitors or mAbs represents an effective strategy to disrupt angiogenic pathways that are fostering the aberrant vasculature. (f) another physical barrier is the extracellular matrix that is composed mainly of collagen. Collagen is produced by TAMs, CAFs, and tumor cells and can impair immune activity through interactions with LAIR-1. Blockade of LAIR-1 combined with PD-1, and TGF-β blockade can increase M1 TAMs population, CD8^+^ T cell infiltration, while decreasing TGF-β and collagen. Figure adapted from J Clin Invest. 2022;132^8^: e155148. https://doi.Org/10.1172/JCI155148. lastly, repolarization of the immunosuppressive microenvironment as an alternative strategy to overcome certain resistant mechanisms can be achieved by (g) the use of mAbs targeting CD33 and TRAIL-R2 receptors expressed in tumor cells were demonstrated to eliminate MDSCs. The use of anti-CFR-1 were demonstrated to eliminate M2 macrophages. Likewise, the depletion of tregs using anti-CD4, anti-CD25 or anti-GITR in combination with ICI have shown to improve CD8+ T cell activity. Another approach uses to decrease the immunosuppressive population that abrogates the effect of ICIs, is the use of small molecule inhibitors targeting CXCR1/2 which are receptors to chemokines essential for the recruitment of MDSCs and TANs. Growing evidence have shown that blocking TGF-b and PD-L1 simultaneously can decrease immunosuppressive population in the TME. (h) the use of inhibitors against IDO enzyme, halt the conversion of tryptophan to kynurenine allowing the increase of NK cells and CD8^+^ T cell and the decrease of Tregs.

ICD: immunogenic cell death; DAMPs: damage-associated molecular patterns; APC: antigen presenting cells; TME: tumor microenvironment; ACT: adoptive cell transfer; TCR T: T cell receptor-engineered T cells; CAR T cells: chimeric antigen receptor T cells; MCH: major histocompatibility complex; VEGF: vascular endothelial growth factor; TAMs: tumor-associated macrophages; LAIR-1: leukocyte-associated immunoglobulin-like receptor-1; CAFs: cancer-associated fibroblasts; TANs: tumor-associated neutrophils; PD-1: program cell-death 1; TGF-β:transforming growth factor beta.

Lack of tumor antigen, defective antigen processing and presentation, and insufficient T cell–DC interaction all contribute to deficient T cell priming and activation that undermines the effect of immunotherapy. Anticancer treatments that induce immunogenic cell death (ICD), such as oncolytic viruses, radiotherapy, and certain types of chemotherapy and small-molecule inhibitors, can bridge this gap. Tumor cells undergoing ICD release antigens and damage-associated molecular patterns (DAMPs), allowing for the recruitment and maturation of APCs, the presentation of targetable antigens to effector T cells, and subsequent induction of antitumor immune responses and immune memory (Figure 2a).^128^ Furthermore, chemotherapy and radiotherapy can upregulate the antigen processing machinery, promote the expression of MHC class I, and elicit the expression of death receptors, rendering the tumor cells that survived the treatment more susceptible to immune attack (Figure 2a).^129,130^ Several preclinical studies, including those on ICI-resistant tumor models, demonstrate that induction of ICD can potentiate the efficacy of ICI therapy.^131–133^ The exact role of ICD is yet to be elucidated in a clinical setting, but growing evidence suggests that ICD inducers may play an important role in the antitumor effect of ICIs.^134–136^ Most inducers of ICD, such as CAR T cells, are not “targeted,” at least not to the same degree as checkpoint blockade; therefore, future studies on the immunotherapy field should focus on developing a consensus on the importance of defining a more generalized approach. Ongoing clinical trials are investigating the safety and the effect of combining ICD-inducers with ICI therapy, including in cancers that are refractory to checkpoint blockade (NCT03474497; Table 1).

### 5.2 Increasing the number of tumor-reactive T cells

Achieving high numbers of infiltrating tumor-reactive T cells is crucial for ICI response. The most direct way to increase the number of effector cells is via adoptive cell transfer (ACT) using TILs, TCR-engineered T cells, or chimeric antigen receptor (CAR)-engineered T cells. ACT with TILs involves isolating lymphocytes from resected tumors, expanding the cells *ex vivo*, and selecting for tumor-reactivity before re-infusing the TILs into patients.^137,138^ In a clinical study, TIL therapy was shown to induce durable responses in patients with metastatic melanoma. Notably, 11 patients enrolled in the study had failed CTLA-4 treatment. Of the 11, five patients experienced complete regression while two experienced partial regression after TIL ACT.^137,138^ TCR T cells are peripheral T cells that are engineered to express tumor antigen-specific TCRs that are modified for improved expression and function.^139^ As such, TCR T cells rely on antigen presentation via MHC molecules but can be generated to target antigenic peptides of intracellular and extracellular origins. Clinical studies using TCR T cells directed against the cancer-testis antigen NY-ESO-1 resulted in promising responses in different tumor types.^140–142^ Preclinical studies combining NY-ESO-1 TCR T cells with ICI demonstrated improved antitumor activity related to increased T cell efficacy (Figure 2b).^143,144^ However, in a pilot trial, the combination of NY-ESO-1 TCR T cell and NY-ESO-1 DC vaccine resulted in transient antitumor response and the addition of ipilimumab to the combination did not improve clinical benefit,^145^ indicating that optimization may be warranted. Clinical trials evaluating the efficacy of TCR T cells with anti-PD-1 or anti-PD-L1 are currently underway.^146^ CAR T cells express receptors composed of an antibody-derived single-chain variable fragment for antigen recognition fused to an intracellular signaling domain derived from T cell signaling proteins.^147^ Contrary to TCR T cells, CAR T cells are MHC-independent and can only target cell surface antigens (Figure 2b). Advancements in this ACT platform have led to six approved CAR T cell therapies for hematological cancers. In solid tumors, however, the efficacy of CAR T cells is severely limited partly due to the immunosuppressive environment that the adoptively transferred cells encounter.^148^ Hence, a great deal of effort is being exerted on studying the application of CAR T cells with ICI. Furthermore, the role of checkpoint blockade in ACT efficacy is paramount; thus, autologous T cells and CAR T cells with knocked out or disrupted PD-1 are being developed and evaluated.^149,150^

Cancer vaccines can amplify a preexisting response by expanding tumor-specific T cell populations, broadening the T cell repertoire, and promoting the transport of T cells to tumor lesions (Figure 2c).^151,152^ Cancer vaccines are typically composed of soluble tumor antigens (e.g., oncoviral, oncofetal, cancer-testis, or neoantigens), formulation (e.g., peptide, nucleic acid, or whole tumor), delivery vehicle (e.g., liposome, cell-based, or viral-based), and an immune adjuvant (e.g., CD40 agonist, TLR agonist, or GM-CSF). Although monotherapy activity of cancer vaccines has so far been limited, its ability to sensitize host immunity to the tumor can complement ICI therapy, and this prospective synergistic activity is the focus of several clinical trials^153^ (Table 1). The phase 1 Lipo-MERIT Trial (NCT02410733) aimed to evaluate the efficacy and safety of a tetravalent RNA vaccine (targeting the tumor antigens NY-ESO-1, MAGE-A3, tyrosinase, and TPTE) with or without PD-1 blockade in metastatic melanoma.^154^ Partial responses were observed in both vaccine monotherapy (12%) and vaccine plus anti-PD-1 treatment (35.3%). Notably, patients with disease that progressed under ICI treatment achieved partial responses with the monotherapy or the combination. Furthermore, the response was associated with the induction of T cells specific to at least one vaccine-targeted antigen and the formation of memory T cells. The data suggest that cancer vaccines may be a strategy to overcome ICI resistance by synergizing with ICI in ICI-experienced tumors.

Immunostimulatory cytokines may also be key combination partners for ICI. One example is IL-12, which is a pro-inflammatory cytokine produced by APCs that promotes Th1 polarization, enhances effector cell recruitment, and boosts CD8^+^ T cell and NK cell cytolytic functions (Figure 2d).^155–157^ Intratumoral administration of IL-12 in conjunction with CTLA-4 blockade has been shown to act synergistically to eradicate murine glioblastoma.^158^ Moreover, in murine tumor models resistant to anti-PD-1/anti-PD-L1 therapy, tumor-targeted NHS-IL12 (IL-12 fused to an antibody that binds exposed DNA commonly found in necrotic tumors) when administered with a histone deacetylase inhibitor potentiated CD8^+^ T cell-dependent antitumor activity and provided survival benefit.^33^ However, a phase 1b study evaluating the combination of NHS-IL12 and avelumab was discontinued due to lack of efficacy (NCT02994953). Several active studies that involve NHS-IL12 with anti-PD-L1/anti-TGF-β fusion protein are ongoing but have yet to post results (NCT04287868, NCT04303117).

Another cytokine that has the potential to elevate ICI activity is IL-15, which plays an important role in the activation, proliferation, survival, and function of NK and cytotoxic CD8+ T cells, as well as the maintenance and survival of memory T cells.^159–161^ In colon and prostate murine tumor models, the dual blockade of CTLA-4 and PD-L1 in combination with recombinant IL-15 (rIL-15) treatment resulted in tumor growth suppression associated with cytotoxic T cell activity, IFNγ secretion, and inhibition of Treg functions.^162,163^ Based on these findings, a phase 1 trial has been designed to test the safety of combining rIL-15 with nivolumab and ipilimumab in patients with refractory cancers (NCT03388632).^160^ Due to the short lifespan of rIL-15 *in vivo*, N803, an IL-15 superagonist composed of IL-15 mutant (IL-15N72D) complexed to a dimeric sushi domain of IL15Rα (IL-15 RαSu) and fused to an IgG-Fc fragment, was developed.^161,164^ The combination of N803 and ICI has been the focus of several preclinical and clinical studies. In syngeneic breast and colon murine tumor models, the combination of N803 and PD-L1 blockade promoted the activation, proliferation, and cytotoxicity of CD8^+^ T cells and NK cells, resulting in decreased tumor burden (Figure 2d).^165^ Meanwhile, in an oral squamous cell carcinoma model, N803 plus PD-1 blockade had moderate therapeutic efficacy that was further enhanced with the adoptive transfer of a PD-L1-targeted CAR-NK.^166^ In the clinic, a completed phase 1/phase 2 study in NSCLC patients demonstrated that N803 and nivolumab were well-tolerated.^167^ Furthermore, six (29%) of the 21 patients enrolled in the study achieved an objective response. Eleven of the 21 patients had relapsed after PD-1 blockade therapy and, of the 11, three (27%) had partial responses and seven (64%) had stable disease. Similarly, an ongoing phase 2b aims to evaluate the safety and efficacy of N803 plus ICI in patients who have progressed after PD-1/PD-L1 treatment (NCT03228667; Table 1). In this trial, patients with disease progression with N803 + ICI roll over to a new cohort and receive N803, PD-1/PD-L1 checkpoint inhibitor, and PD-L1-targeted CAR-NK.

The pathophysiological properties of tumors result in physical barriers that prevent the infiltration of effector cells. One impediment is the tumor vasculature, which is characterized by abnormal endothelial cells, chaotic vessel growth, leakiness, and erratic blood flow.^168^ Tumor vascular endothelial cells also express the death ligand FasL, killing extravasating CD8^+^ T cells while sparing Tregs that are not as sensitive to Fas-mediated apoptosis.^169^ Furthermore, VEGF, the critical driver of angiogenesis, promotes an immunosuppressive ecosystem in the TME.^170^ The first anti-angiogenic drug, bevacizumab, an anti-VEGFmAb, was initially approved in 2004 as a treatment for colorectal cancer in combination with chemotherapy.^171,172^ Since then, research has been conducted to improve patient response to bevacizumab and other antiangiogenic drugs, mainly monoclonal antibodies and small-molecule tyrosine kinase inhibitors that target VEGF/VEGFR and other angiogenic pathways, in combination with other anti-cancer agents.^173^ Several murine tumor models posit that the combination of ICI with antiangiogenic agents results in better antitumor effects, associated with normalized vasculature, increased T cell infiltration, improved antigen presentation, and decreased immunosuppressive cell populations (Figure 2e).^174–176^ The combination of ICI with antiangiogenic agents has also achieved success in the clinic. For instance, the open-label, randomized phase 3 IMbrave150 (NCT03434379) study in patients with unresectable hepatocellular carcinoma showed that atezolizumab, an anti-PD-L1 antibody, with bevacizumab resulted in superior overall and progression-free survival outcomes than standard-of-care sorafenib, a multikinase inhibitor.^177,178^ This trial informed the FDA approval of this combination in hepatocellular carcinoma in 2020.^179^ In addition, a phase 1b clinical trial (NCT02715531) demonstrated that in patients with unresectable hepatocellular carcinoma, atezolizumab plus bevacizumab significantly prolonged progression-free survival compared with atezolizumab monotherapy.^180^ Collectively, these studies show that the combination of ICI with an antiangiogenic agent results in improved clinical benefit when compared to monotherapy with either treatment.

*The extracellular matrix* is another physical barrier that effector immune cells must overcome to effectively attack tumor cells. In addition to regulating the migration of T cells, collagen produced by cancer-associated fibroblasts, macrophages, and tumor cells can impair immune activity through interactions with leukocyte-associated immunoglobulin-like receptor-1 (LAIR-1).^181^ LAIR-1 activation and signaling on T cells, NK cells, monocytes, and DCs result in the inhibition of these immune cells.^32,182–184^ An experimental antibody, NC410, that competes with LAIR-1 for collagen-binding is currently being evaluated in a phase 1 study in patients with advanced and metastatic solid tumors, such as ovarian, gastric, and colorectal cancer (NCT04408599). In murine EMT6 breast and MC38 colon cancer models, the combination of NC410 and an anti-PD-L1/anti-TGF-β fusion protein antibody (bintrafusp alfa, formerly M7824) improved antitumor activity by remodeling the collagen matrix, enhancing T cell tumor infiltration, and skewing the tumor-associated macrophages from the immunosuppressive M2 phenotype to immune-favorable M1 (Figure 2f).^32^ This preclinical study underscores the valuable contribution of targeting the extracellular matrix in facilitating ICI therapy.

### 5.3 Repolarization of the immunosuppressive microenvironment

The immunosuppressive tumor microenvironment is comprised of cellular components and soluble factors that promote tumor progression and contribute to immune resistance. The abundance of myeloid-derived suppressor cells (MDSCs), Tregs, TAMs, and other immunosuppressive cells in the peripheral blood or in the tumor lesion has been associated with poor prognosis in cancer patients.^185^ Immunosuppressive cells, together with tumor cells and stromal cells, can produce inhibitory cytokines (e.g., IL-10 and TGF-b) and factors (e.g., arginase, IDO, and collagen) and express checkpoint ligands to dampen the immune response.^185,186^

Several immuno-oncology agents that deplete or reprogram immunosuppressive populations by targeting markers overexpressed on those cells are under development and have the potential to synergize with ICIs. Antibodies that target CD33 (gemtuzumab ozogamicin, BI 8,366,858) and TRAIL-R2 (DS-8273a) were demonstrated to eliminate MDSCs (Figure 2g).^187–189^ A phase 1 trial studying the combination of DS-8273a and nivolumab has recently been completed, but results are yet to be published (NCT02983006). Likewise, targeting colony stimulating factor-1 receptor (CSF-1 R), which regulates monocyte migration, proliferation, and differentiation into TAMs, has been shown to reduce TAM populations (Figure 2g).^190^ The safety and efficacy of axatilimab, an anti-CSF1-R antibody, in combination with nivolumab in patients with refractory/relapsed classical Hodgkin lymphoma is the subject of an ongoing phase 2 trial (NCT05723055, Table 1). Targeting MARCO, which is expressed on immunosuppressive M2-like TAMs, reprogrammed the macrophages into a pro-inflammatory phenotype and enhanced the antitumor efficacy of anti-CTLA-4 in melanoma and colon carcinoma models.^191^ Studies on mouse models also suggest that depletion of Tregs using anti-CD4, anti-CD25, or anti-GITR (glucocorticoid-induced tumor-necrosis-factor receptor (TNFR)-related protein) antibodies in combination with ICI improves CD8+ T cell activity, resulting in control of tumor growth (Figure 2g).^192–194^ However, several phase 1 trials demonstrated that anti-GITR (MK-4166, MK-1248, or TRX518) in combination with anti-PD-1 (pembrolizumab or nivolumab) only resulted in limited clinical responses.^195–197^ A study investigating the safety and efficacy of another anti-GITR mAb, REGN6569, plus PD-1 blockade with cemiplimab is underway with results pending (NCT04465487). However, based on what is currently known, additional work will be required to translate the benefits of anti-GITR and ICI combination into an effective therapy.^198^

Blocking the recruitment and migration of immunosuppressive cells can also effectively repolarize the TME and sensitize tumors to ICI. For example, targeting CXCR1 and CXCR2, which are receptors to chemokines essential for MDSC and tumor-associated neutrophil (TAN) recruitment, can prevent MDSC and TAN accumulation, enhance effector cell function, and augment the antitumor activity of PD-1/PD-L1 blockade in preclinical models (Figure 2g).^199–202^ SX-682, a small molecule dual inhibitor of CXCR1 and CXCR2, is currently being investigated in combination with anti-PD-1 (nivolumab, pembrolizumab, or tislelizumab) in patients with colorectal cancer (NCT04599140), melanoma (NCT03161431; Table 1), pancreatic cancer (NCT05604560), and non-small cell lung cancer (NCT05570825).

Lastly, another potential strategy to re-invigorate effector cells in tandem with ICIs is to target immunosuppressive factors in the TME. IDO is an enzyme involved in tryptophan catabolism, converting tryptophan to kynurenine. In cancer settings, the depletion of tryptophan and accumulation of kynurenine promotes Treg activation and inhibits NK and CD8+ T cell activation, thereby resulting in immunosuppression (Figure 2g).^203,204^ The IDO inhibitor, epacadostat, showed promising anti-tumor activity when combined with pembrolizumab and nivolumab in phase 1/2 trials.^205^ However, a phase 3 study of epacadostat plus pembrolizumab did not improve progression-free or overall survival in patients with unresectable or metastatic melanoma.^205,206^ Another metabolite that accumulates in the TME and plays an important role in immunosuppression is extracellular adenosine (eADO). The adenosine pathway ultimately results in the conversion of extracellular ATP to eADO via ectonucleotidases CD39 and CD73, which are overexpressed in tumors.^207^ Ligation of eADO to adenosine receptors (A2_A_ and/or A2_B_) on effector cells results in the reduction in CD8^+^ T cell function, differentiation of naïve CD4^+^ T cells to Treg cells, and decreased NK proliferation, survival, and function.^123^ Immuno-oncology agents targeting CD39, CD73, and adenosine and their potential combinations have been reviewed recently by Zahavi and Hodge (Figure 2h).^123^ Cytokines that support the immunosuppressive milieu can also be targeted to enhance ICI activity. TGF-β is expressed by tumor cells, stromal cells, and immunosuppressive cells that can attenuate CD8^+^ T cell function,^208,209^ inhibit CD8^+^ T cell infiltration,^210^ expand Tregs,^211^ and polarize neutrophils into pro-tumor populations.^212^ The value of simultaneous blockade of PD-1/PD-L1 axis and TGF-β is underscored by the development of bintrafusp alfa, which is a fusion antibody composed of an anti-PD-L1 antibody fused to the extracellular domain of human TGF-β receptor II (Figure 2g). In preclinical studies, monotherapy with bintrafusp alfa was shown to effectively trap TGF-β and bind PD-L1, resulting in decreased tumor burden and prolonged survival.^213,214^ Furthermore, treatment with bintrafusp alfa had superior anti-tumor activity than treatment with either anti-PD-L1 or TGF-β, highlighting the importance of co-targeting these two inhibitory molecules. A recent publication by Gameiro et al. reviews past and current clinical trials involving bintrafusp alfa.^215^

## Conclusion

To date, the benefits of cancer immunotherapy are limited to certain cancer types, mainly because of the heterogenicity intrinsic to different tumor types and to the mechanisms promoting primary and acquired resistance. Efforts made in the field to overcome CBR-related unresponsiveness to ICI therapy have been focussed on overcoming T cell-related deficiencies and/or suppressing immunosuppressive populations in the TME. However, additional effort is needed to close the gap in knowledge in identifying additional checkpoints that may be abrogating the effectiveness of ICI therapy as well as unknown mechanisms underlying CBR. More detailed guidelines are needed as well to follow mixed responses after ICI therapy and to define primary and acquired resistance more accurately, not only in the setting of a clinical trial but also in the real-world after agent approval.

## References

[1] Sharma P, Hu-Lieskovan S, Wargo JA, Ribas A. Primary, adaptive, and acquired resistance to cancer immunotherapy. Cell. 2017;168(4):707–20. doi:10.1016/j.cell.2017.01.017. 28187290 PMC5391692

[2] Marin-Acevedo JA, Kimbrough EO, Lou Y. Next generation of immune checkpoint inhibitors and beyond. J Hematol Oncol. 2021;14(1):45. doi:10.1186/s13045-021-01056-8. 33741032 PMC7977302

[3] Santiago-Sanchez GS, Hodge JW, Fabian KP. Tipping the scales: immunotherapeutic strategies that disrupt immunosuppression and promote immune activation. Front Immunol. 2022;13:993624. doi:10.3389/fimmu.2022.993624. 36159809 PMC9492957

[4] McDermott D, Haanen J, Chen TT, Lorigan P, O’Day S, investigators MDX. Efficacy and safety of ipilimumab in metastatic melanoma patients surviving more than 2 years following treatment in a phase III trial (MDX010-20). Ann Oncol. 2013;24(10):2694–2698. doi:10.1093/annonc/mdt291. 23942774

[5] Brunet J-F, Denizot F, Luciani M-F, Roux-Dosseto M, Suzan M, Mattei M-G, Golstein P. A new member of the immunoglobulin superfamily—CTLA-4. Nature. 1987;328(6127):267–270. doi:10.1038/328267a0. 3496540

[6] Sansom DM. CD28, CTLA-4 and their ligands: who does what and to whom? Immunology. 2000;101(2):169–77. doi:10.1046/j.1365-2567.2000.00121.x. 11012769 PMC2327073

[7] Egen JG, Kuhns MS, Allison JP. CTLA-4: new insights into its biological function and use in tumor immunotherapy. Nat Immunol. 2002;3(7):611–8. doi:10.1038/ni0702-611. 12087419

[8] Walker LSK, Sansom DM. Confusing signals: recent progress in CTLA-4 biology. Trends Immunol. 2015;36(2):63–70. doi:10.1016/j.it.2014.12.001. 25582039 PMC4323153

[9] Sansom DM, Walker LSK. The role of CD28 and cytotoxic T-lymphocyte antigen-4 (CTLA-4) in regulatory T-cell biology. Immunol Rev. 2006;212(1):131–48. doi:10.1111/j.0105-2896.2006.00419.x. 16903911

[10] Hurst JH. Cancer immunotherapy innovator James Allison receives the 2015 Lasker~DeBakey clinical medical research award. J Clin Invest. 2015;125(10):3732–6. doi:10.1172/JCI84236. 26345422 PMC4607124

[11] Schadendorf D, Hodi FS, Robert C, Weber JS, Margolin K, Hamid O, Patt D, Chen T-T, Berman DM, Wolchok JD, et al. Pooled analysis of long-term survival data from Phase II and Phase III trials of ipilimumab in unresectable or metastatic melanoma. J Clin Oncol. 2015;33(17):1889–94. doi:10.1200/JCO.2014.56.2736. 25667295 PMC5089162

[12] Vaddepally RK, Kharel P, Pandey R, Garje R, Chandra AB. Review of Indications of FDA-Approved immune checkpoint inhibitors per NCCN Guidelines With The Level Of Evidence. Cancers Basel. 2020;12(3):738. doi:10.3390/cancers12030738. 32245016 PMC7140028

[13] Dalle S, Mortier L, Corrie P, Lotem M, Board R, Arance AM, Meiss F, Terheyden P, Gutzmer R, Buysse B, et al. Long-term real-world experience with ipilimumab and non-ipilimumab therapies in advanced melanoma: the IMAGE study. BMC Cancer. 2021;21(1):642. doi:10.1186/s12885-021-08032-y. 34051732 PMC8164785

[14] Cramer-van der Welle CM, Verschueren MV, Tonn M, Peters BJM, Schramel F, Klungel OH, Groen HJM, van de Garde EMW, Kastelijn EA, Vermeer LC, et al. Real-world outcomes versus clinical trial results of immunotherapy in stage IV non-small cell lung cancer (NSCLC) in the Netherlands. Sci Rep. 2021;11(1):6306. doi:10.1038/s41598-021-85696-3. 33737641 PMC7973789

[15] Weber JS, D’Angelo SP, Minor D, Hodi FS, Gutzmer R, Neyns B, Hoeller C, Khushalani NI, Miller WH, Lao CD, et al. Nivolumab versus chemotherapy in patients with advanced melanoma who progressed after anti-CTLA-4 treatment (CheckMate 037): a randomised, controlled, open-label, phase 3 trial. Lancet Oncol. 2015;16(4):375–84. doi:10.1016/S1470-2045(15)70076-8. 25795410

[16] Kaufman HL, Russell J, Hamid O, Bhatia S, Terheyden P, D’Angelo SP, Shih KC, Lebbé C, Linette GP, Milella M, et al. Avelumab in patients with chemotherapy-refractory metastatic merkel cell carcinoma: a multicentre, single-group, open-label, phase 2 trial. Lancet Oncol. 2016;17(10):1374–85. doi:10.1016/S1470-2045(16)30364-3. 27592805 PMC5587154

[17] Keilholz U, Mehnert JM, Bauer S, Bourgeois H, Patel MR, Gravenor D, Nemunaitis JJ, Taylor MH, Wyrwicz L, Lee K-W, et al. Avelumab in patients with previously treated metastatic melanoma: phase 1b results from the JAVELIN solid tumor trial. J Immunother Cancer. 2019;7(1):12. doi:10.1186/s40425-018-0459-y. 30651126 PMC6335739

[18] Massard C, Gordon MS, Sharma S, Rafii S, Wainberg ZA, Luke J, Curiel TJ, Colon-Otero G, Hamid O, Sanborn RE, et al. Safety and efficacy of durvalumab (MEDI4736), an anti–programmed cell death ligand-1 immune checkpoint inhibitor, in patients with advanced urothelial bladder cancer. J Clin Oncol. 2016;34(26):3119–3125. doi:10.1200/JCO.2016.67.9761. 27269937 PMC5569690

[19] Hodi FS, O’Day SJ, McDermott DF, Weber RW, Sosman JA, Haanen JB, Gonzalez R, Robert C, Schadendorf D, Hassel JC, et al. Improved survival with ipilimumab in patients with metastatic melanoma. N Engl J Med. 2010;363(8):711–23. doi:10.1056/NEJMoa1003466. 20525992 PMC3549297

[20] Larkin J, Chiarion-Sileni V, Gonzalez R, Grob JJ, Cowey CL, Lao CD, Schadendorf D, Dummer R, Smylie M, Rutkowski P, et al. Combined nivolumab and ipilimumab or monotherapy in untreated melanoma. N Engl J Med. 2015;373(1):23–34. doi:10.1056/NEJMoa1504030. 26027431 PMC5698905

[21] Schachter J, Ribas A, Long GV, Arance A, Grob JJ, Mortier L, Daud A, Carlino MS, McNeil C, Lotem M, et al. Pembrolizumab versus ipilimumab for advanced melanoma: final overall survival results of a multicentre, randomised, open-label phase 3 study (KEYNOTE-006). Lancet. 2017;390(10105):1853–62. doi:10.1016/S0140-6736(17)31601-X. 28822576

[22] van Elsas MJ, van Hall T, van der Burg SH. Future challenges in cancer resistance to immunotherapy. Cancers Basel. 2020;12(4):935. doi:10.3390/cancers12040935. 32290124 PMC7226490

[23] Kluger HM, Tawbi HA, Ascierto ML, Bowden M, Callahan MK, Cha E, Chen HX, Drake CG, Feltquate DM, Ferris RL, et al. Defining tumor resistance to PD-1 pathway blockade: recommendations from the first meeting of the SITC immunotherapy resistance taskforce. J Immunother Cancer. 2020;8(1):e000398. doi:10.1136/jitc-2019-000398. 32238470 PMC7174063

[24] De Martino M, Vanpouille-Box C, Galluzzi L. Immunological barriers to immunotherapy in primary and metastatic breast cancer. EMBO Mol Med. 2021;13(8):e14393. doi:10.15252/emmm.202114393. 34128586 PMC8350896

[25] Emens LA. Breast cancer immunotherapy: facts and hopes. Clin Cancer Res. 2018;24(3):511–20. doi:10.1158/1078-0432.CCR-16-3001. 28801472 PMC5796849

[26] Vukadin S, Khaznadar F, Kizivat T, Vcev A, Smolic M. Molecular mechanisms of resistance to immune checkpoint inhibitors in melanoma treatment: an update. Biomedicines. 2021;9(7):835. doi:10.3390/biomedicines9070835. 34356899 PMC8301472

[27] Boyero L, Sanchez-Gastaldo A, Alonso M, Noguera-Ucles JF, Molina-Pinelo S, Bernabe-Caro R. Primary and acquired resistance to immunotherapy in lung cancer: unveiling the mechanisms underlying of immune checkpoint blockade therapy. Cancers Basel. 2020;12(12):3729. doi:10.3390/cancers12123729. 33322522 PMC7763130

[28] Walsh RJ, Soo RA. Resistance to immune checkpoint inhibitors in non-small cell lung cancer: biomarkers and therapeutic strategies. Ther Adv Med Oncol. 2020;12:1758835920937902. doi:10.1177/1758835920937902. 32670423 PMC7339077

[29] Tang T, Huang X, Zhang G, Hong Z, Bai X, Liang T. Advantages of targeting the tumor immune microenvironment over blocking immune checkpoint in cancer immunotherapy. Signal Transduct Target Ther. 2021;6(1):72. doi:10.1038/s41392-020-00449-4. 33608497 PMC7896069

[30] Fabian KP, Padget MR, Fujii R, Schlom J, Hodge JW. Differential combination immunotherapy requirements for inflamed (warm) tumors versus T cell excluded (cool) tumors: engage, expand, enable, and evolve. J Immunother Cancer. 2021;9(2):e001691. doi:10.1136/jitc-2020-001691. 33602696 PMC7896589

[31] Hicks KC, Chariou PL, Ozawa Y, Minnar CM, Knudson KM, Meyer TJ, Bian J, Cam M, Schlom J, Gameiro SR, et al. Tumour-targeted interleukin-12 and entinostat combination therapy improves cancer survival by reprogramming the tumour immune cell landscape. Nat Commun. 2021;12(1):5151. doi:10.1038/s41467-021-25393-x. 34446712 PMC8390765

[32] Horn LA, Chariou PL, Gameiro SR, Qin H, Iida M, Fousek K, Meyer TJ, Cam M, Flies D, Langermann S, et al. Remodeling the tumor microenvironment via blockade of LAIR-1 and TGF-β signaling enables PD-L1–mediated tumor eradication. J Clin Invest. 2022;132(8). doi:10.1172/JCI155148. PMC9012291 35230974

[33] Minnar CM, Chariou PL, Horn LA, Hicks KC, Palena C, Schlom J, Gameiro SR. Tumor-targeted interleukin-12 synergizes with entinostat to overcome PD-1/PD-L1 blockade-resistant tumors harboring MHC-I and APM deficiencies. J Immunother Cancer. 2022;10(6):e004561. doi:10.1136/jitc-2022-004561. 35764364 PMC9240938

[34] Holmgaard RB, Zamarin D, Munn DH, Wolchok JD, Allison JP. Indoleamine 2,3-dioxygenase is a critical resistance mechanism in antitumor T cell immunotherapy targeting CTLA-4. J Exp Med. 2013;210(7):1389–402. doi:10.1084/jem.20130066. 23752227 PMC3698523

[35] Franks SE, Santiago-Sanchez GS, Fabian KP, Solocinski K, Chariou PL, Hamilton DH, Kowalczyk JT, Padget MR, Gameiro SR, Schlom J, et al. Exploiting docetaxel-induced tumor cell necrosis with tumor targeted delivery of IL-12. Cancer Immunol Immunother. 2023;72(8):2783–2797. doi:10.1007/s00262-023-03459-7. 37166485 PMC10361896

[36] Ribas A, Dummer R, Puzanov I, VanderWalde A, Andtbacka RHI, Michielin O, Olszanski AJ, Malvehy J, Cebon J, Fernandez E, et al. Oncolytic virotherapy promotes intratumoral T cell infiltration and improves Anti-PD-1 immunotherapy. Cell. 2017;170(6):1109–1119.e10. doi:10.1016/j.cell.2017.08.027. 28886381 PMC8034392

[37] Fabian KP, Malamas AS, Padget MR, Solocinski K, Wolfson B, Fujii R, Abdul Sater H, Schlom J, Hodge JW. Therapy of established tumors with rationally designed multiple agents targeting diverse immune–tumor interactions: engage, expand, enable. Cancer Immunol Res. 2021;9(2):239–252. doi:10.1158/2326-6066.CIR-20-0638. 33355290 PMC7864895

[38] Maleki Vareki S. High and low mutational burden tumors versus immunologically hot and cold tumors and response to immune checkpoint inhibitors. J Immunother Cancer. 2018;6(1):157. doi:10.1186/s40425-018-0479-7. 30587233 PMC6307306

[39] O’Donnell JS, Long GV, Scolyer RA, Teng MW, Smyth MJ. Resistance to PD1/PDL1 checkpoint inhibition. Cancer Treat Rev. 2017;52:71–81. doi:10.1016/j.ctrv.2016.11.007. 27951441

[40] Sahin IH, Akce M, Alese O, Shaib W, Lesinski GB, El-Rayes B, Wu C. Immune checkpoint inhibitors for the treatment of MSI-H/MMR-D colorectal cancer and a perspective on resistance mechanisms. Br J Cancer. 2019;121(10):809–818. doi:10.1038/s41416-019-0599-y. 31607751 PMC6889302

[41] Luciano S, Haudenschild C, Kuranz S. Using real world data to examine outcomes in immunotherapy-treated patients with metastatic non-small cell lung cancer. J Clin Oncol. 2020;38(15_suppl):e21715–e. doi:10.1200/JCO.2020.38.15_suppl.e21715.

[42] Miao K, Zhang X, Wang H, Si X, Ni J, Zhong W, Zhao J, Xu Y, Chen M, Pan R, et al. Real-world data of different immune checkpoint inhibitors for non-small cell lung cancer in China. Front Oncol. 2022;12:859938. doi:10.3389/fonc.2022.859938. 35392244 PMC8982065

[43] Velcheti V, Hu X, Yang L, Pietanza MC, Burke T. Long-term real-world outcomes of first-line pembrolizumab monotherapy for metastatic non-small cell lung cancer with >/=50% expression of programmed cell death-ligand 1. Front Oncol. 2022;12:834761. doi:10.3389/fonc.2022.834761. 35402266 PMC8990758

[44] Kehl KL, Greenwald S, Chamoun NG, Manberg PJ, Schrag D. Association between first-line immune checkpoint inhibition and survival for medicare-insured patients with advanced non-small cell lung cancer. JAMA Netw Open. 2021;4(5):e2111113. doi:10.1001/jamanetworkopen.2021.11113. 34019086 PMC8140374

[45] Eroglu Z, Zaretsky JM, Hu-Lieskovan S, Kim DW, Algazi A, Johnson DB, Liniker E, Kong B, Munhoz R, Rapisuwon S, et al. High response rate to PD-1 blockade in desmoplastic melanomas. Nature. 2018;553(7688):347–50. doi:10.1038/nature25187. 29320474 PMC5773412

[46] Rose AAN, Armstrong SM, Hogg D, Butler MO, Saibil SD, Arteaga DP, Pimentel Muniz T, Kelly D, Ghazarian D, King I, et al. Biologic subtypes of melanoma predict survival benefit of combination anti-PD1+anti-CTLA4 immune checkpoint inhibitors versus anti-PD1 monotherapy. J Immunother Cancer. 2021;9(1):e001642. doi:10.1136/jitc-2020-001642. 33483342 PMC7831745

[47] Stege HM, Haist M, Schultheis S, Fleischer MI, Mohr P, Ugurel S, Terheyden P, Thiem A, Kiecker F, Leiter U, et al. Response durability after cessation of immune checkpoint inhibitors in patients with metastatic merkel cell carcinoma: a retrospective multicenter DeCOG study. Cancer Immunol Immunother. 2021;70(11):3313–22. doi:10.1007/s00262-021-02925-4. 33870464 PMC8505278

[48] Zaggana E, Konstantinou MP, Krasagakis GH, de Bree E, Kalpakis K, Mavroudis D, Krasagakis K. Merkel Cell Carcinoma—Update on Diagnosis, Management and Future Perspectives. Cancers Basel. 2022;15(1):103. doi:10.3390/cancers15010103. 36612102 PMC9817518

[49] Galon J, Bruni D. Approaches to treat immune hot, altered and cold tumours with combination immunotherapies. Nat Rev Drug Discov. 2019;18(3):197–218. doi:10.1038/s41573-018-0007-y. 30610226

[50] Duan Q, Zhang H, Zheng J, Zhang L. Turning cold into hot: firing up the tumor microenvironment. Trends Cancer. 2020;6(7):605–18. doi:10.1016/j.trecan.2020.02.022. 32610070

[51] Knight A, Karapetyan L, Kirkwood JM. Immunotherapy in melanoma: recent advances and future directions. Cancers Basel. 2023;15(4):1106. doi:10.3390/cancers15041106. 36831449 PMC9954703

[52] LoPiccolo J, Schollenberger MD, Dakhil S, Rosner S, Ali O, Sharfman WH, Silk AW, Bhatia S, Lipson EJ. Rescue therapy for patients with anti-PD-1-refractory merkel cell carcinoma: a multicenter, retrospective case series. J Immunother Cancer. 2019;7(1):170. doi:10.1186/s40425-019-0661-6. 31287031 PMC6615256

[53] Cowey CL, Mahnke L, Espirito J, Helwig C, Oksen D, Bharmal M. Real-world treatment outcomes in patients with metastatic merkel cell carcinoma treated with chemotherapy in the USA. Future Oncol. 2017;13(19):1699–1710. doi:10.2217/fon-2017-0187. 28605939

[54] D’Angelo SP, Russell J, Lebbe C, Chmielowski B, Gambichler T, Grob JJ, Kiecker F, Rabinowits G, Terheyden P, Zwiener I, et al. Efficacy and safety of first-line avelumab treatment in patients with stage iv metastatic merkel cell carcinoma: a preplanned interim analysis of a clinical trial. JAMA Oncol. 2018;4(9):e180077. doi:10.1001/jamaoncol.2018.0077. 29566106 PMC5885245

[55] Nghiem PT, Bhatia S, Lipson EJ, Kudchadkar RR, Miller NJ, Annamalai L, Berry S, Chartash EK, Daud A, Fling SP, et al. PD-1 blockade with pembrolizumab in advanced merkel-cell carcinoma. N Engl J Med. 2016;374(26):2542–52. doi:10.1056/NEJMoa1603702. 27093365 PMC4927341

[56] Weppler AM, Da Meda L, Pires da Silva I, Xu W, Grignani G, Menzies AM, Carlino MS, Long GV, Lo SN, Nordman I, et al. Durability of response to immune checkpoint inhibitors in metastatic Merkel cell carcinoma after treatment cessation. Eur J Cancer. 2023;183:109–18. doi:10.1016/j.ejca.2023.01.016. 36842413

[57] Reck M, Rodriguez-Abreu D, Robinson AG, Hui R, Csoszi T, Fulop A, Gottfried M, Peled N, Tafreshi A, Cuffe S, et al. Pembrolizumab versus chemotherapy for PD-L1–positive non–small-cell lung cancer. N Engl J Med. 2016;375(19):1823–1833. doi:10.1056/NEJMoa1606774. 27718847

[58] Burtness B, Harrington KJ, Greil R, Soulieres D, Tahara M, de Castro G Jr., Psyrri A, Basté N, Neupane P, Bratland Å, et al. Pembrolizumab alone or with chemotherapy versus cetuximab with chemotherapy for recurrent or metastatic squamous cell carcinoma of the head and neck (KEYNOTE-048): a randomised, open-label, phase 3 study. Lancet. 2019;394(10212):1915–1928. doi:10.1016/S0140-6736(19)32591-7. 31679945

[59] Janjigian YY, Shitara K, Moehler M, Garrido M, Salman P, Shen L, Wyrwicz L, Yamaguchi K, Skoczylas T, Campos Bragagnoli A, et al. First-line nivolumab plus chemotherapy versus chemotherapy alone for advanced gastric, gastro-oesophageal junction, and oesophageal adenocarcinoma (CheckMate 649): a randomised, open-label, phase 3 trial. Lancet. 2021;398(10294):27–40. doi:10.1016/S0140-6736(21)00797-2. 34102137 PMC8436782

[60] Schmid P, Adams S, Rugo HS, Schneeweiss A, Barrios CH, Iwata H, Diéras V, Hegg R, Im S-A, Shaw Wright G, et al. Atezolizumab and Nab-Paclitaxel in Advanced Triple-Negative Breast Cancer. N Engl J Med. 2018;379(22):2108–21. doi:10.1056/NEJMoa1809615. 30345906

[61] Sun JM, Shen L, Shah MA, Enzinger P, Adenis A, Doi T, Kojima T, Metges J-P, Li Z, Kim S-B, et al. Pembrolizumab plus chemotherapy versus chemotherapy alone for first-line treatment of advanced oesophageal cancer (KEYNOTE-590): a randomised, placebo-controlled, phase 3 study. Lancet. 2021;398(10302):759–71. doi:10.1016/S0140-6736(21)01234-4. 34454674

[62] Garassino MC, Gadgeel S, Esteban E, Felip E, Speranza G, Domine M, Hochmair MJ, Powell S, Cheng SYS, Bischoff HG, et al. Patient-reported outcomes following pembrolizumab or placebo plus pemetrexed and platinum in patients with previously untreated, metastatic, non-squamous non-small-cell lung cancer (KEYNOTE-189): a multicentre, double-blind, randomised, placebo-controlled, phase 3 trial. Lancet Oncol. 2020;21(3):387–97. doi:10.1016/S1470-2045(19)30801-0. 32035514

[63] Lanka SM, Zorko NA, Antonarakis ES, Barata PC. Metastatic Castration-Resistant Prostate Cancer, Immune Checkpoint Inhibitors, and Beyond. Curr Oncol. 2023;30(4):4246–4256. doi:10.3390/curroncol30040323. 37185436 PMC10137248

[64] Anagnostou V, Smith KN, Forde PM, Niknafs N, Bhattacharya R, White J, Zhang T, Adleff V, Phallen J, Wali N, et al. Evolution of neoantigen landscape during immune checkpoint blockade in non–small cell lung cancer. Cancer Discov. 2017;7(3):264–276. doi:10.1158/2159-8290.CD-16-0828. 28031159 PMC5733805

[65] Le DT, Durham JN, Smith KN, Wang H, Bartlett BR, Aulakh LK, Lu S, Kemberling H, Wilt C, Luber BS, et al. Mismatch repair deficiency predicts response of solid tumors to PD-1 blockade. Sci. 2017;357(6349):409–13. doi:10.1126/science.aan6733. PMC5576142 28596308

[66] Zhou B, Gao Y, Zhang P, Chu Q. Acquired resistance to immune checkpoint blockades: the underlying mechanisms and potential strategies. Front Immunol. 2021;12:693609. doi:10.3389/fimmu.2021.693609. 34194441 PMC8236848

[67] Schwartz LH, Litiere S, de Vries E, Ford R, Gwyther S, Mandrekar S, Shankar L, Bogaerts J, Chen A, Dancey J, et al. RECIST 1.1—update and clarification: from the RECIST committee. Eur J Cancer. 2016;62:132–137. doi:10.1016/j.ejca.2016.03.081. 27189322 PMC5737828

[68] Seymour L, Bogaerts J, Perrone A, Ford R, Schwartz LH, Mandrekar S, Lin NU, Litière S, Dancey J, Chen A, et al. iRECIST: guidelines for response criteria for use in trials testing immunotherapeutics. Lancet Oncol. 2017;18(3):e143–e152. doi:10.1016/S1470-2045(17)30074-8. 28271869 PMC5648544

[69] Ramon-Patino JL, Schmid S, Lau S, Seymour L, Gaudreau PO, Li JJN, Bradbury PA, Calvo E. iRECIST and atypical patterns of response to immuno-oncology drugs. J Immunother Cancer. 2022;10(6):e004849. doi:10.1136/jitc-2022-004849. 35715004 PMC9207898

[70] Chiou VL, Burotto M. Pseudoprogression and immune-related response in solid tumors. J Clin Oncol. 2015;33(31):3541–3. doi:10.1200/JCO.2015.61.6870. 26261262 PMC4622096

[71] Wolchok JD, Hoos A, O’Day S, Weber JS, Hamid O, Lebbe C, Maio M, Binder M, Bohnsack O, Nichol G, et al. Guidelines for the evaluation of immune therapy activity in solid tumors: immune-related response criteria. Clin Cancer Res. 2009;15(23):7412–20. doi:10.1158/1078-0432.CCR-09-1624. 19934295

[72] Ma Y, Wang Q, Dong Q, Zhan L, Zhang J. How to differentiate pseudoprogression from true progression in cancer patients treated with immunotherapy. Am J Cancer Res. 2019;9(8):1546–53. 31497342 PMC6726978

[73] Hodi FS, Hwu WJ, Kefford R, Weber JS, Daud A, Hamid O, Patnaik A, Ribas A, Robert C, Gangadhar TC, et al. Evaluation of immune-related response criteria and RECIST v1.1 in patients with advanced melanoma treated with pembrolizumab. J Clin Oncol. 2016;34(13):1510–7. doi:10.1200/JCO.2015.64.0391. 26951310 PMC5070547

[74] Cai Y, Tian Y, Wang J, Wei W, Tang Q, Lu L, Luo Z, Li W, Lu Y, Pu J, et al. Identification of driver genes regulating the T-Cell–infiltrating levels in hepatocellular carcinoma. Front Genet. 2020;11:560546. doi:10.3389/fgene.2020.560546. 33381145 PMC7767976

[75] Wang H, Guo M, Wei H, Chen Y. Targeting p53 pathways: mechanisms, structures, and advances in therapy. Signal Transduct Target Ther. 2023;8(1):92. doi:10.1038/s41392-023-01347-1. 36859359 PMC9977964

[76] Sprooten J, De Wijngaert P, Vanmeerbeerk I, Martin S, Vangheluwe P, Schlenner S, Krysko DV, Parys JB, Bultynck G, Vandenabeele P, et al. Necroptosis in immuno-oncology and cancer immunotherapy. Cells. 2020;9(8):1823. doi:10.3390/cells9081823. 32752206 PMC7464343

[77] Jenkins RW, Barbie DA, Flaherty KT. Mechanisms of resistance to immune checkpoint inhibitors. Br J Cancer. 2018;118(1):9–16. doi:10.1038/bjc.2017.434. 29319049 PMC5765236

[78] Adachi K, Davis MM. T-cell receptor ligation induces distinct signaling pathways in naive vs. antigen-experienced T cells. Proc Natl Acad Sci U S A. 2011;108(4):1549–1554. doi:10.1073/pnas.1017340108. 21205892 PMC3029746

[79] Ngan HL, Law CH, Choi YCY, Chan JY, Lui VWY. Precision drugging of the MAPK pathway in head and neck cancer. NPJ Genom Med. 2022;7(1):20. doi:10.1038/s41525-022-00293-1. 35296678 PMC8927572

[80] Ngan HL, Liu Y, Fong AY, Poon PHY, Yeung CK, Chan SSM, Lau A, Piao W, Li H, Tse JSW, et al. MAPK pathway mutations in head and neck cancer affect immune microenvironments and ErbB3 signaling. Life Sci Alli. 2020;3(6):e201900545. doi:10.26508/lsa.201900545. PMC7219112 32381551

[81] Peng W, Chen JQ, Liu C, Malu S, Creasy C, Tetzlaff MT, Xu C, McKenzie JA, Zhang C, Liang X, et al. Loss of PTEN promotes resistance to T cell–mediated immunotherapy. Cancer Discov. 2016;6(2):202–216. doi:10.1158/2159-8290.CD-15-0283. 26645196 PMC4744499

[82] Spranger S, Bao R, Gajewski TF. Melanoma-intrinsic beta-catenin signalling prevents anti-tumour immunity. Nature. 2015;523(7559):231–235. doi:10.1038/nature14404. 25970248

[83] Tentler JJ, Lang J, Capasso A, Kim DJ, Benaim E, Lee YB, Eisen A, Bagby SM, Hartman SJ, Yacob BW, et al. RX-5902, a novel β-catenin modulator, potentiates the efficacy of immune checkpoint inhibitors in preclinical models of triple-negative breast cancer. BMC Cancer. 2020;20(1):1063. doi:10.1186/s12885-020-07500-1. 33148223 PMC7641792

[84] Jorgovanovic D, Song M, Wang L, Zhang Y. Roles of IFN-γ in tumor progression and regression: a review. Biomark Res. 2020;8(1):49. doi:10.1186/s40364-020-00228-x. 33005420 PMC7526126

[85] Gao J, Shi LZ, Zhao H, Chen J, Xiong L, He Q, Chen T, Roszik J, Bernatchez C, Woodman SE, et al. Loss of IFN-γ pathway genes in tumor cells as a mechanism of resistance to anti-CTLA-4 therapy. Cell. 2016;167(2):397–404.e9. doi:10.1016/j.cell.2016.08.069. 27667683 PMC5088716

[86] Zaretsky JM, Garcia-Diaz A, Shin DS, Escuin-Ordinas H, Hugo W, Hu-Lieskovan S, Torrejon DY, Abril-Rodriguez G, Sandoval S, Barthly L, et al. Mutations associated with acquired resistance to PD-1 blockade in melanoma. N Engl J Med. 2016;375(9):819–29. doi:10.1056/NEJMoa1604958. 27433843 PMC5007206

[87] Ribas A, Hamid O, Daud A, Hodi FS, Wolchok JD, Kefford R, Joshua AM, Patnaik A, Hwu W-J, Weber JS, et al. Association of pembrolizumab with tumor response and survival among patients with advanced melanoma. JAMA. 2016;315(15):1600–9. doi:10.1001/jama.2016.4059. 27092830

[88] Blum JS, Wearsch PA, Cresswell P. Pathways of antigen processing. Annu Rev Immunol. 2013;31(1):443–73. doi:10.1146/annurev-immunol-032712-095910. 23298205 PMC4026165

[89] Mpakali A, Stratikos E. The role of antigen processing and presentation in cancer and the efficacy of immune checkpoint inhibitor immunotherapy. Cancers Basel. 2021;13(1):134. doi:10.3390/cancers13010134. 33406696 PMC7796214

[90] Sha D, Jin Z, Budczies J, Kluck K, Stenzinger A, Sinicrope FA. Tumor mutational burden as a predictive biomarker in solid tumors. Cancer Discov. 2020;10(12):1808–25. doi:10.1158/2159-8290.CD-20-0522. 33139244 PMC7710563

[91] Jardim DL, Goodman A, de Melo Gagliato D, Kurzrock R. The challenges of tumor mutational burden as an immunotherapy biomarker. Cancer Cell. 2021;39(2):154–73. doi:10.1016/j.ccell.2020.10.001. 33125859 PMC7878292

[92] Shao C, Li G, Huang L, Pruitt S, Castellanos E, Frampton G, Carson KR, Snow T, Singal G, Fabrizio D, et al. Prevalence of high tumor mutational burden and association with survival in patients with less common solid tumors. JAMA Netw Open. 2020;3(10):e2025109. doi:10.1001/jamanetworkopen.2020.25109. 33119110 PMC7596577

[93] Zheng M. Tumor mutation burden for predicting immune checkpoint blockade response: the more, the better. J Immunother Cancer. 2022;10(1):e003087. doi:10.1136/jitc-2021-003087. 35101940 PMC8804687

[94] Hodi FS, Wolchok JD, Schadendorf D, Larkin J, Long GV, Qian X, Saci A, Young TC, Srinivasan S, Chang H, et al. TMB and inflammatory gene expression associated with clinical outcomes following immunotherapy in advanced melanoma. Cancer Immunol Res. 2021;9(10):1202–1213. doi:10.1158/2326-6066.CIR-20-0983. 34389558 PMC9414280

[95] Goodman AM, Kato S, Bazhenova L, Patel SP, Frampton GM, Miller V, Stephens PJ, Daniels GA, Kurzrock R. Tumor mutational burden as an independent predictor of response to immunotherapy in diverse cancers. Mol Cancer Ther. 2017;16(11):2598–2608. doi:10.1158/1535-7163.MCT-17-0386. 28835386 PMC5670009

[96] Imamura T, Ashida R, Ohshima K, Uesaka K, Sugiura T, Ohgi K, Yamada M, Otsuka S, Hatakeyama K, Nagashima T, et al. Characterization of pancreatic cancer with ultra-low tumor mutational burden. Sci Rep. 2023;13(1):4359. doi:10.1038/s41598-023-31579-8. 36928600 PMC10020557

[97] Thomas R, Al-Khadairi G, Decock J. Immune Checkpoint Inhibitors in Triple Negative Breast Cancer Treatment: Promising Future Prospects. Front Oncol. 2020;10:600573. doi:10.3389/fonc.2020.600573. 33718107 PMC7947906

[98] ElTanbouly MA, Croteau W, Noelle RJ, Lines JL. VISTA: a novel immunotherapy target for normalizing innate and adaptive immunity. Semin Immunol. 2019;42:101308. doi:10.1016/j.smim.2019.101308. 31604531 PMC7233310

[99] Kato S, Okamura R, Kumaki Y, Ikeda S, Nikanjam M, Eskander R, Goodman A, Lee S, Glenn ST, Dressman D, et al. Expression of TIM3/VISTA checkpoints and the CD68 macrophage-associated marker correlates with anti-PD1/PDL1 resistance: implications of immunogram heterogeneity. Oncoimmunology. 2020;9(1):1708065. doi:10.1080/2162402X.2019.1708065. 32117584 PMC7028323

[100] Topalian SL, Drake CG, Pardoll DM. Immune checkpoint blockade: a common denominator approach to cancer therapy. Cancer Cell. 2015;27(4):450–61. doi:10.1016/j.ccell.2015.03.001. 25858804 PMC4400238

[101] Mulati K, Hamanishi J, Matsumura N, Chamoto K, Mise N, Abiko K, Baba T, Yamaguchi K, Horikawa N, Murakami R, et al. VISTA expressed in tumour cells regulates T cell function. Br J Cancer. 2019;120(1):115–27. doi:10.1038/s41416-018-0313-5. 30382166 PMC6325144

[102] Wang L, Rubinstein R, Lines JL, Wasiuk A, Ahonen C, Guo Y, Lu L-F, Gondek D, Wang Y, Fava RA, et al. VISTA, a novel mouse Ig superfamily ligand that negatively regulates T cell responses. J Exp Med. 2011;208(3):577–92. doi:10.1084/jem.20100619. 21383057 PMC3058578

[103] Kakavand H, Jackett LA, Menzies AM, Gide TN, Carlino MS, Saw RPM, Thompson JF, Wilmott JS, Long GV, Scolyer RA, et al. Negative immune checkpoint regulation by VISTA: a mechanism of acquired resistance to anti-PD-1 therapy in metastatic melanoma patients. Mod Pathol. 2017;30(12):1666–76. doi:10.1038/modpathol.2017.89. 28776578

[104] Li HB, Yang ZH, Guo QQ. Immune checkpoint inhibition for pancreatic ductal adenocarcinoma: limitations and prospects: a systematic review. Cell Commun Signal. 2021;19(1):117. doi:10.1186/s12964-021-00789-w. 34819086 PMC8611916

[105] Johnson DB, Estrada MV, Salgado R, Sanchez V, Doxie DB, Opalenik SR, Vilgelm AE, Feld E, Johnson AS, Greenplate AR, et al. Melanoma-specific MHC-II expression represents a tumour-autonomous phenotype and predicts response to anti-PD-1/PD-L1 therapy. Nat Commun. 2016;7(1):10582. doi:10.1038/ncomms10582. 26822383 PMC4740184

[106] Johnson DB, Nixon MJ, Wang Y, Wang DY, Castellanos E, Estrada MV, Ericsson-Gonzalez PI, Cote CH, Salgado R, Sanchez V, et al. Tumor-specific MHC-II expression drives a unique pattern of resistance to immunotherapy via LAG-3/FCRL6 engagement. JCI Insight. 2018;3(24). doi:10.1172/jci.insight.120360. PMC6338319 30568030

[107] Matsuzaki J, Gnjatic S, Mhawech-Fauceglia P, Beck A, Miller A, Tsuji T, Eppolito C, Qian F, Lele S, Shrikant P, et al. Tumor-infiltrating NY-ESO-1–specific CD8+T cells are negatively regulated by LAG-3 and PD-1 in human ovarian cancer. Proc Natl Acad Sci U S A. 2010;107(17):7875–7880. doi:10.1073/pnas.1003345107. 20385810 PMC2867907

[108] Andrews LP, Cillo AR, Karapetyan L, Kirkwood JM, Workman CJ, Vignali DAA. Molecular pathways and mechanisms of LAG3 in cancer therapy. Clin Cancer Res. 2022;28(23):5030–9. doi:10.1158/1078-0432.CCR-21-2390. 35579997 PMC9669281

[109] Draghi A, Chamberlain CA, Furness A, Donia M. Acquired resistance to cancer immunotherapy. Semin Immunopathol. 2019;41(1):31–40. doi:10.1007/s00281-018-0692-y. 29968044

[110] Koyama S, Akbay EA, Li YY, Herter-Sprie GS, Buczkowski KA, Richards WG, Gandhi L, Redig AJ, Rodig SJ, Asahina H, et al. Adaptive resistance to therapeutic PD-1 blockade is associated with upregulation of alternative immune checkpoints. Nat Commun. 2016;7(1):10501. doi:10.1038/ncomms10501. 26883990 PMC4757784

[111] Shayan G, Srivastava R, Li J, Schmitt N, Kane LP, Ferris RL. Adaptive resistance to anti-PD1 therapy by tim-3 upregulation is mediated by the PI3K-Akt pathway in head and neck cancer. Oncoimmunology. 2017;6(1):e1261779. doi:10.1080/2162402X.2016.1261779. 28197389 PMC5283618

[112] Sakuishi K, Apetoh L, Sullivan JM, Blazar BR, Kuchroo VK, Anderson AC. Targeting tim-3 and PD-1 pathways to reverse T cell exhaustion and restore anti-tumor immunity. J Exp Med. 2010;207(10):2187–94. doi:10.1084/jem.20100643. 20819927 PMC2947065

[113] Franks SE, Fabian KP, Santiago-Sanchez G, Wolfson B, Hodge JW. Immune targeting of three independent suppressive pathways (TIGIT, PD-L1, TGFbeta) provides significant antitumor efficacy in immune checkpoint resistant models. Oncoimmunology. 2022;11(1):2124666. doi:10.1080/2162402X.2022.2124666. 36211806 PMC9542338

[114] Ge Z, Peppelenbosch MP, Sprengers D, Kwekkeboom J. TIGIT, the next step towards successful combination immune checkpoint therapy in cancer. Front Immunol. 2021;12:699895. doi:10.3389/fimmu.2021.699895. 34367161 PMC8339559

[115] Kawashima S, Inozume T, Kawazu M, Ueno T, Nagasaki J, Tanji E, Honobe A, Ohnuma T, Kawamura T, Umeda Y, et al. TIGIT/CD155 axis mediates resistance to immunotherapy in patients with melanoma with the inflamed tumor microenvironment. J Immunother Cancer. 2021;9(11):e003134. doi:10.1136/jitc-2021-003134. 34795004 PMC8603290

[116] Freed-Pastor WA, Lambert LJ, Ely ZA, Pattada NB, Bhutkar A, Eng G, Mercer KL, Garcia AP, Lin L, Rideout WM, et al. The CD155/TIGIT axis promotes and maintains immune evasion in neoantigen-expressing pancreatic cancer. Cancer Cell. 2021;39(10):1342–1360.e14. doi:10.1016/j.ccell.2021.07.007. 34358448 PMC8511341

[117] Zhulai G, Oleinik E. Targeting regulatory T cells in anti-PD-1/PD-L1 cancer immunotherapy. Scand J Immunol. 2022;95(3):e13129. doi:10.1111/sji.13129. 34936125

[118] Batlle E, Massague J. Transforming growth factor-beta signaling in immunity and cancer. Immunity. 2019;50(4):924–940. doi:10.1016/j.immuni.2019.03.024. 30995507 PMC7507121

[119] Ni Y, Soliman A, Joehlin-Price A, Rose PG, Vlad A, Edwards RP, Mahdi H. High TGF-β signature predicts immunotherapy resistance in gynecologic cancer patients treated with immune checkpoint inhibition. Npj Precis Oncol. 2021;5(1):101. doi:10.1038/s41698-021-00242-8. 34921236 PMC8683510

[120] Martin CJ, Datta A, Littlefield C, Kalra A, Chapron C, Wawersik S, Dagbay KB, Brueckner CT, Nikiforov A, Danehy FT, et al. Selective inhibition of TGFβ1 activation overcomes primary resistance to checkpoint blockade therapy by altering tumor immune landscape. Sci Transl Med. 2020;12(536). doi:10.1126/scitranslmed.aay8456. 32213632

[121] Siewe N, Friedman A, Najbauer J. TGF-β inhibition can overcome cancer primary resistance to PD-1 blockade: a mathematical model. PloS ONE. 2021;16(6):e0252620. doi:10.1371/journal.pone.0252620. 34061898 PMC8168900

[122] Fares CM, Van Allen EM, Drake CG, Allison JP, Hu-Lieskovan S. Mechanisms of resistance to immune checkpoint blockade: why does checkpoint inhibitor immunotherapy not work for all patients? Am Soc Clin Oncol Educ Book. 2019;39(39):147–164. doi:10.1200/EDBK_240837. 31099674

[123] Zahavi D, Hodge JW. Targeting Immunosuppressive Adenosine Signaling: a Review of Potential Immunotherapy Combination Strategies. Int J Mol Sci. 2023;24(10):8871. doi:10.3390/ijms24108871. 37240219 PMC10218801

[124] Fujiwara Y, Kato S, Nesline MK, Conroy JM, DePietro P, Pabla S, Kurzrock R. Indoleamine 2,3-dioxygenase (IDO) inhibitors and cancer immunotherapy. Cancer Treat Rev. 2022;110:102461. doi:10.1016/j.ctrv.2022.102461. 36058143 PMC12187009

[125] Beasley GM, Brown MC, Farrow NE, Landa K, Al-Rohil RN, Selim MA, Therien AD, Jung S-H, Gao J, Boczkowski D, et al. Multimodality analysis confers a prognostic benefit of a T-cell infiltrated tumor microenvironment and peripheral immune status in patients with melanoma. J Immunother Cancer. 2022;10(9):e005052. doi:10.1136/jitc-2022-005052. 36175036 PMC9528663

[126] Sullivan RJ, Gutierrez M, Khattak A, Thomas SS, Jimeno A, Pascarella S, Pascarella S, Zhu L, Morrissey M, Meehan RS, et al. Phase 1/2 study of mRNA-4359 administered alone and in combination with immune checkpoint blockade in adult participants with advanced solid tumors. J Clin Oncol. 2023;41(16_suppl):TPS2676–TPS. doi:10.1200/JCO.2023.41.16_suppl.TPS2676.

[127] Jost M, Chen Y, Gilbert LA, Horlbeck MA, Krenning L, Menchon G, Rai A, Cho MY, Stern JJ, Prota AE, et al. Pharmaceutical-grade rigosertib is a microtubule-destabilizing agent. Mol Cell. 2020;79(1):191–198.e3. doi:10.1016/j.molcel.2020.06.008. 32619469 PMC7332992

[128] Kroemer G, Galassi C, Zitvogel L, Galluzzi L. Immunogenic cell stress and death. Nat Immunol. 2022;23(4):487–500. doi:10.1038/s41590-022-01132-2. 35145297

[129] Fabian KP, Wolfson B, Hodge JW. From Immunogenic Cell Death to Immunogenic Modulation: Select Chemotherapy Regimens Induce a Spectrum of Immune-Enhancing Activities in the Tumor Microenvironment. Front Oncol. 2021;11:728018. doi:10.3389/fonc.2021.728018. 34497771 PMC8419351

[130] Fabian KP, Kowalczyk JT, Reynolds ST, Hodge JW. Dying of stress: chemotherapy, radiotherapy, and small-molecule inhibitors in immunogenic cell death and immunogenic modulation. Cells. 2022;11(23):3826. doi:10.3390/cells11233826. 36497086 PMC9737874

[131] Pfirschke C, Engblom C, Rickelt S, Cortez-Retamozo V, Garris C, Pucci F, Yamazaki T, Poirier-Colame V, Newton A, Redouane Y, et al. Immunogenic Chemotherapy Sensitizes Tumors to Checkpoint Blockade Therapy. Immunity. 2016;44(2):343–54. doi:10.1016/j.immuni.2015.11.024. 26872698 PMC4758865

[132] Yamazaki T, Buqué A, Ames TD, Galluzzi L. PT-112 induces immunogenic cell death and synergizes with immune checkpoint blockers in mouse tumor models. OncoImmunology. 2020;9(1):1721810. doi:10.1080/2162402X.2020.1721810. 32117585 PMC7028345

[133] Zhou H, Tu C, Yang P, Li J, Kepp O, Li H, Zhang L, Zhang L, Zhao Y, Zhang T, et al. Carbon ion radiotherapy triggers immunogenic cell death and sensitizes melanoma to anti-PD-1 therapy in mice. Oncoimmunology. 2022;11(1):2057892. doi:10.1080/2162402X.2022.2057892. 35355680 PMC8959514

[134] Kepp O, Zitvogel L, Kroemer G. Clinical evidence that immunogenic cell death sensitizes to PD-1/PD-L1 blockade. Oncoimmunology. 2019;8(10):e1637188. doi:10.1080/2162402X.2019.1637188. 31646079 PMC6791413

[135] Mansfield AS, Każarnowicz A, Karaseva N, Sánchez A, De Boer R, Andric Z, Reck M, Atagi S, Lee J-S, Garassino M, et al. Safety and patient-reported outcomes of atezolizumab, carboplatin, and etoposide in extensive-stage small-cell lung cancer (IMpower133): a randomized phase I/III trial. Ann Oncol. 2020;31(2):310–7. doi:10.1016/j.annonc.2019.10.021. 31959349

[136] Røssevold AH, Andresen NK, Bjerre CA, Gilje B, Jakobsen EH, Raj SX, Falk RS, Russnes HG, Jahr T, Mathiesen RR, et al. Atezolizumab plus anthracycline-based chemotherapy in metastatic triple-negative breast cancer: the randomized, double-blind phase 2b ALICE trial. Nat Med. 2022;28(12):2573–83. doi:10.1038/s41591-022-02126-1. 36482103 PMC9800277

[137] Rosenberg SA, Restifo NP. Adoptive cell transfer as personalized immunotherapy for human cancer. Sci. 2015;348(6230):62–8. doi:10.1126/science.aaa4967. PMC6295668 25838374

[138] Rosenberg SA, Yang JC, Sherry RM, Kammula US, Hughes MS, Phan GQ, Citrin DE, Restifo NP, Robbins PF, Wunderlich JR, et al. Durable complete responses in heavily pretreated patients with metastatic melanoma using T-cell transfer immunotherapy. Clin Cancer Res. 2011;17(13):4550–7. doi:10.1158/1078-0432.CCR-11-0116. 21498393 PMC3131487

[139] Zhao Q, Jiang Y, Xiang S, Kaboli PJ, Shen J, Zhao Y, Wu X, Du F, Li M, Cho CH, et al. Engineered TCR-T cell immunotherapy in anticancer precision medicine: pros and cons. Front Immunol. 2021;12:12. doi:10.3389/fimmu.2021.658753. PMC8042275 33859650

[140] Rapoport AP, Stadtmauer EA, Binder-Scholl GK, Goloubeva O, Vogl DT, Lacey SF, Badros AZ, Garfall A, Weiss B, Finklestein J, et al. NY-ESO-1–specific TCR–engineered T cells mediate sustained antigen-specific antitumor effects in myeloma. Nat Med. 2015;21(8):914–21. doi:10.1038/nm.3910. 26193344 PMC4529359

[141] Robbins PF, Morgan RA, Feldman SA, Yang JC, Sherry RM, Dudley ME, Wunderlich JR, Nahvi AV, Helman LJ, Mackall CL, et al. Tumor regression in patients with metastatic synovial cell sarcoma and melanoma using genetically engineered lymphocytes reactive with NY-ESO-1. J Clin Oncol. 2011;29(7):917–24. doi:10.1200/JCO.2010.32.2537. 21282551 PMC3068063

[142] Robbins PF, Kassim SH, Tran TL, Crystal JS, Morgan RA, Feldman SA, Yang JC, Dudley ME, Wunderlich JR, Sherry RM, et al. A pilot trial using lymphocytes genetically engineered with an NY-ESO-1–reactive T-cell receptor: long-term follow-up and correlates with response. Clin Cancer Res. 2015;21(5):1019–1027. doi:10.1158/1078-0432.CCR-14-2708. 25538264 PMC4361810

[143] Moon EK, Ranganathan R, Eruslanov E, Kim S, Newick K, O’Brien S, Lo A, Liu X, Zhao Y, Albelda SM, et al. Blockade of programmed death 1 augments the ability of human t cells engineered to target NY-ESO-1 to control tumor growth after adoptive transfer. Clin Cancer Res. 2016;22(2):436–47. doi:10.1158/1078-0432.CCR-15-1070. 26324743 PMC4715990

[144] Martinez M, Kim S, St Jean N, O’Brien S, Lian L, Sun J, Verona RI, Moon E. Addition of anti-TIM3 or anti-TIGIT antibodies to anti-PD1 blockade augments human T cell adoptive cell transfer. Oncoimmunology. 2021;10(1):1873607. doi:10.1080/2162402X.2021.1873607. 33537176 PMC7833767

[145] Nowicki TS, Berent-Maoz B, Cheung-Lau G, Huang RR, Wang X, Tsoi J, Kaplan-Lefko P, Cabrera P, Tran J, Pang J, et al. A pilot trial of the combination of transgenic NY-ESO-1–reactive adoptive cellular therapy with dendritic cell vaccination with or without ipilimumab. Clin Cancer Res. 2019;25(7):2096–108. doi:10.1158/1078-0432.CCR-18-3496. 30573690 PMC6445780

[146] Rossetti R, Brand H, Lima SCG, Furtado IP, Silveira RM, Fantacini DMC, Covas DT, de Souza LEB. Combination of genetically engineered T cells and immune checkpoint blockade for the treatment of cancer. Immunol Adv. 2022;2(1):ltac005. doi:10.1093/immadv/ltac005. PMC9327125 35919489

[147] Mitra A, Barua A, Huang L, Ganguly S, Feng Q, He B. From bench to bedside: the history and progress of CAR T cell therapy. Front Immunol. 2023;14:1188049. doi:10.3389/fimmu.2023.1188049. 37256141 PMC10225594

[148] Marofi F, Motavalli R, Safonov VA, Thangavelu L, Yumashev AV, Alexander M, Shomali N, Chartrand MS, Pathak Y, Jarahian M, et al. CAR T cells in solid tumors: challenges and opportunities. Stem Cell Res Ther. 2021;12(1):81. doi:10.1186/s13287-020-02128-1. 33494834 PMC7831265

[149] McGowan E, Lin Q, Ma G, Yin H, Chen S, Lin Y. PD-1 disrupted CAR-T cells in the treatment of solid tumors: promises and challenges. Biomed Pharmacother. 2020;121:109625. doi:10.1016/j.biopha.2019.109625. 31733578

[150] Xu Y, Chen C, Guo Y, Hu S, Sun Z. 2022. Effect of CRISPR/Cas9-Edited PD-1/PD-L1 on Tumor Immunity and Immunotherapy. Front Immunol. 13:848327. 10.3389/fimmu.2022.848327. 35300341 PMC8920996

[151] Ott PA, Wu CJ. Cancer vaccines: steering T cells down the right path to eradicate tumors. Cancer Discov. 2019;9(4):476–81. doi:10.1158/2159-8290.CD-18-1357. 30862723 PMC7067230

[152] Hu Z, Ott PA, Wu CJ. Towards personalized, tumour-specific, therapeutic vaccines for cancer. Nat Rev Immunol. 2018;18(3):168–82. doi:10.1038/nri.2017.131. 29226910 PMC6508552

[153] Wolfson B, Franks SE, Hodge JW. Stay on target: reengaging cancer vaccines in combination immunotherapy. Vaccines (Basel). 2021;9(5):509. doi:10.3390/vaccines9050509. 34063388 PMC8156017

[154] Sahin U, Oehm P, Derhovanessian E, Jabulowsky RA, Vormehr M, Gold M, Maurus D, Schwarck-Kokarakis D, Kuhn AN, Omokoko T, et al. An RNA vaccine drives immunity in checkpoint-inhibitor-treated melanoma. Nature. 2020;585(7823):107–12. doi:10.1038/s41586-020-2537-9. 32728218

[155] Colombo MP, Trinchieri G. Interleukin-12 in anti-tumor immunity and immunotherapy. Cytokine Growth Factor Rev. 2002;13(2):155–68. doi:10.1016/S1359-6101(01)00032-6. 11900991

[156] Mirlekar B, Pylayeva-Gupta Y. IL-12 family cytokines in cancer and immunotherapy. Cancers Basel. 2021;13(2):167. doi:10.3390/cancers13020167. 33418929 PMC7825035

[157] Tugues S, Burkhard SH, Ohs I, Vrohlings M, Nussbaum K, Vom Berg J, Kulig P, Becher B. New insights into IL-12-mediated tumor suppression. Cell Death Differ. 2015;22(2):237–246. doi:10.1038/cdd.2014.134. 25190142 PMC4291488

[158] Vom Berg J, Vrohlings M, Haller S, Haimovici A, Kulig P, Sledzinska A, Weller M, Becher B. Intratumoral IL-12 combined with CTLA-4 blockade elicits T cell–mediated glioma rejection. J Exp Med. 2013;210(13):2803–2811. doi:10.1084/jem.20130678. 24277150 PMC3865478

[159] Waldmann TA. The biology of interleukin-2 and interleukin-15: implications for cancer therapy and vaccine design. Nat Rev Immunol. 2006;6(8):595–601. doi:10.1038/nri1901. 16868550

[160] Waldmann TA, Dubois S, Miljkovic MD, Conlon KC. IL-15 in the combination immunotherapy of cancer. Front Immunol. 2020;11:11. doi:10.3389/fimmu.2020.00868. 32508818 PMC7248178

[161] Robinson TO, Schluns KS. The potential and promise of IL-15 in immuno-oncogenic therapies. Immunol Lett. 2017;190:159–68. doi:10.1016/j.imlet.2017.08.010. 28823521 PMC5774016

[162] Yu P, Steel JC, Zhang M, Morris JC, Waldmann TA. Simultaneous blockade of multiple immune system inhibitory checkpoints enhances antitumor activity mediated by interleukin-15 in a murine metastatic colon carcinoma model. Clin Cancer Res. 2010;16(24):6019–28. doi:10.1158/1078-0432.CCR-10-1966. 20924130 PMC3005104

[163] Yu P, Steel JC, Zhang M, Morris JC, Waitz R, Fasso M, Allison JP, Waldmann TA. Simultaneous inhibition of two regulatory T-cell subsets enhanced Interleukin-15 efficacy in a prostate tumor model. Proc Natl Acad Sci USA. 2012;109(16):6187–6192. doi:10.1073/pnas.1203479109. 22474386 PMC3341063

[164] Lui G, Minnar CM, Soon-Shiong P, Schlom J, Gameiro SR. Exploiting an interleukin-15 heterodimeric agonist (n803) for effective immunotherapy of solid malignancies. Cells. 2023;12(12):1611. doi:10.3390/cells12121611. 37371081 PMC10297013

[165] Knudson KM, Hicks KC, Alter S, Schlom J, Gameiro SR. Mechanisms involved in IL-15 superagonist enhancement of anti-PD-L1 therapy. J Immunother Cancer. 2019;7(1):82. doi:10.1186/s40425-019-0551-y. 30898149 PMC6429734

[166] Fabian KP, Padget MR, Donahue RN, Solocinski K, Robbins Y, Allen CT, Lee JH, Rabizadeh S, Soon-Shiong P, Schlom J, et al. PD-L1 targeting high-affinity NK (t-haNK) cells induce direct antitumor effects and target suppressive MDSC populations. J Immunother Cancer. 2020;8(1):e000450. doi:10.1136/jitc-2019-000450. 32439799 PMC7247398

[167] Wrangle JM, Velcheti V, Patel MR, Garrett-Mayer E, Hill EG, Ravenel JG, Miller JS, Farhad M, Anderton K, Lindsey K, et al. ALT-803, an IL-15 superagonist, in combination with nivolumab in patients with metastatic non-small cell lung cancer: a non-randomised, open-label, phase 1b trial. Lancet Oncol. 2018;19(5):694–704. doi:10.1016/S1470-2045(18)30148-7. 29628312 PMC6089612

[168] Hanahan D, Weinberg Robert A. Hallmarks of cancer: the next generation. Cell. 2011;144(5):646–674. doi:10.1016/j.cell.2011.02.013. 21376230

[169] Motz GT, Santoro SP, Wang LP, Garrabrant T, Lastra RR, Hagemann IS, Lal P, Feldman MD, Benencia F, Coukos G, et al. Tumor endothelium FasL establishes a selective immune barrier promoting tolerance in tumors. Nat Med. 2014;20(6):607–15. doi:10.1038/nm.3541. 24793239 PMC4060245

[170] Patel SA, Nilsson MB, Le X, Cascone T, Jain RK, Heymach JV. Molecular mechanisms and future implications of VEGF/VEGFR in cancer therapy. Clin Cancer Res. 2023;29(1):30–9. doi:10.1158/1078-0432.CCR-22-1366. 35969170 PMC10274152

[171] Hurwitz H, Fehrenbacher L, Novotny W, Cartwright T, Hainsworth J, Heim W, Berlin J, Baron A, Griffing S, Holmgren E, et al. Bevacizumab plus irinotecan, fluorouracil, and leucovorin for metastatic colorectal cancer. N Engl J Med. 2004;350(23):2335–42. doi:10.1056/NEJMoa032691. 15175435

[172] Botrel TEA, Clark L, Paladini L, Clark OAC. Efficacy and safety of bevacizumab plus chemotherapy compared to chemotherapy alone in previously untreated advanced or metastatic colorectal cancer: a systematic review and meta-analysis. BMC Cancer. 2016;16(1):677. doi:10.1186/s12885-016-2734-y. 27558497 PMC4997727

[173] Liu Z-L, Chen H-H, Zheng L-L, Sun L-P, Shi L. Angiogenic signaling pathways and anti-angiogenic therapy for cancer. Sig Transduct Target Ther. 2023;8(1):198. doi:10.1038/s41392-023-01460-1. PMC10175505 37169756

[174] Du Four S, Maenhout SK, Niclou SP, Thielemans K, Neyns B, Aerts JL. Combined VEGFR and CTLA-4 blockade increases the antigen-presenting function of intratumoral DCs and reduces the suppressive capacity of intratumoral MDSCs. Am J Cancer Res. 2016;6(11):2514–2531. doi:10.1080/2162402X.2014.998107. 27904768 PMC5126270

[175] Schmittnaegel M, Rigamonti N, Kadioglu E, Cassará A, Wyser Rmili C, Kiialainen A, Kienast Y, Mueller H-J, Ooi C-H, Laoui D, et al. Dual angiopoietin-2 and VEGFA inhibition elicits antitumor immunity that is enhanced by PD-1 checkpoint blockade. Sci Transl Med. 2017;9(385):eaak9670. doi:10.1126/scitranslmed.aak9670. 28404865

[176] Di Tacchio M, Macas J, Weissenberger J, Sommer K, Bähr O, Steinbach JP, Senft C, Seifert V, Glas M, Herrlinger U, et al. Tumor vessel normalization, immunostimulatory reprogramming, and improved survival in glioblastoma with combined inhibition of PD-1, angiopoietin-2, and VEGF. Cancer Immunol Res. 2019;7(12):1910–27. doi:10.1158/2326-6066.CIR-18-0865. 31597643

[177] Finn RS, Qin S, Ikeda M, Galle PR, Ducreux M, Kim T-Y, Kudo M, Breder V, Merle P, Kaseb AO, et al. Atezolizumab plus bevacizumab in unresectable hepatocellular carcinoma. N Engl J Med. 2020;382(20):1894–905. doi:10.1056/NEJMoa1915745. 32402160

[178] Cheng AL, Qin S, Ikeda M, Galle PR, Ducreux M, Kim TY, Lim HY, Kudo M, Breder V, Merle P, et al. Updated efficacy and safety data from IMbrave150: Atezolizumab plus bevacizumab vs. sorafenib for unresectable hepatocellular carcinoma. J Hepatol. 2022;76(4):862–73. doi:10.1016/j.jhep.2021.11.030. 34902530

[179] Koh B, Tan DJH, Lim WH, Wong JSL, Ng CH, Chan KE, Wang M, Yong WP, Dan YY, Wang LZ, et al. Trial watch: immunotherapeutic strategies on the horizon for hepatocellular carcinoma. OncoImmunology. 2023;12(1):2214478. doi:10.1080/2162402X.2023.2214478. 37284696 PMC10241000

[180] Lee MS, Ryoo B-Y, Hsu C-H, Numata K, Stein S, Verret W, Hack SP, Spahn J, Liu B, Abdullah H, et al. Atezolizumab with or without bevacizumab in unresectable hepatocellular carcinoma (GO30140): an open-label, multicentre, phase 1b study. Lancet Oncol. 2020;21(6):808–20. doi:10.1016/S1470-2045(20)30156-X. 32502443

[181] Rygiel TP, Stolte EH, de Ruiter T, van de Weijer ML, Meyaard L. Tumor-expressed collagens can modulate immune cell function through the inhibitory collagen receptor LAIR-1. Mol Immunol. 2011;49(1–2):402–406. doi:10.1016/j.molimm.2011.09.006. 21955987

[182] Carvalheiro T, Garcia S, Pascoal Ramos MI, Giovannone B, Radstake TRDJ, Marut W, Meyaard L. Leukocyte Associated Immunoglobulin Like Receptor 1 Regulation and Function on Monocytes and Dendritic Cells During Inflammation. Front Immunol. 2020;11:11. doi:10.3389/fimmu.2020.01793. 32973751 PMC7466540

[183] Ramos MIP, Tian L, de Ruiter EJ, Song C, Paucarmayta A, Singh A, Elshof E, Vijver SV, Shaik J, Bosiacki J, et al. Cancer immunotherapy by NC410, a LAIR-2 Fc protein blocking human LAIR-collagen interaction. eLife. 2021;10:e62927. doi:10.7554/eLife.62927. 34121658 PMC8225389

[184] Santiago-Sánchez GS, Hodge JW, Fabian KP. 2022. Tipping the scales: Immunotherapeutic strategies that disrupt immunosuppression and promote immune activation. Front Immunol. 13. 10.3389/fimmu.2022.993624. PMC9492957 36159809

[185] Tie Y, Tang F, Wei Y-Q, Wei X-W. Immunosuppressive cells in cancer: mechanisms and potential therapeutic targets. J Hematol Oncol. 2022;15(1):61. doi:10.1186/s13045-022-01282-8. 35585567 PMC9118588

[186] Burkholder B, Huang R-Y, Burgess R, Luo S, Jones VS, Zhang W, Lv Z-Q, Gao C-Y, Wang B-L, Zhang Y-M, et al. Tumor-induced perturbations of cytokines and immune cell networks. Biochimica Et Biophysica Acta (BBA) - Rev Cancer. 2014;1845(2):182–201. doi:10.1016/j.bbcan.2014.01.004. 24440852

[187] Fultang L, Panetti S, Ng M, Collins P, Graef S, Rizkalla N, Booth S, Lenton R, Noyvert B, Shannon-Lowe C, et al. MDSC targeting with gemtuzumab ozogamicin restores T cell immunity and immunotherapy against cancers. EBioMedicine. 2019;47:235–46. doi:10.1016/j.ebiom.2019.08.025. 31462392 PMC6796554

[188] Dominguez GA, Condamine T, Mony S, Hashimoto A, Wang F, Liu Q, Forero A, Bendell J, Witt R, Hockstein N, et al. Selective targeting of myeloid-derived suppressor cells in cancer patients using DS-8273a, an agonistic TRAIL-R2 antibody. Clin Cancer Res. 2017;23(12):2942–50. doi:10.1158/1078-0432.CCR-16-1784. 27965309 PMC5468499

[189] Eksioglu EA, Chen X, Heider KH, Rueter B, McGraw KL, Basiorka AA, Wei M, Burnette A, Cheng P, Lancet J, et al. Novel therapeutic approach to improve hematopoiesis in low risk MDS by targeting MDSCs with the Fc-engineered CD33 antibody BI 836858. Leukemia. 2017;31(10):2172–80. doi:10.1038/leu.2017.21. 28096534 PMC5552472

[190] Cassetta L, Pollard JW. Targeting macrophages: therapeutic approaches in cancer. Nat Rev Drug Discov. 2018;17(12):887–904. doi:10.1038/nrd.2018.169. 30361552

[191] Georgoudaki A-M, Prokopec Kajsa K, Boura Vanessa V, Hellqvist E, Sohn S, Östling J, Dahan R, Harris R, Rantalainen M, Klevebring D, et al. Reprogramming tumor-associated macrophages by antibody targeting inhibits cancer progression and metastasis. Cell Rep. 2016;15(9):2000–2011. doi:10.1016/j.celrep.2016.04.084. 27210762

[192] Arce Vargas F, Furness AJS, Solomon I, Joshi K, Mekkaoui L, Lesko MH, Miranda Rota E, Dahan R, Georgiou A, Sledzinska A, et al. Fc-optimized anti-CD25 depletes tumor-infiltrating regulatory T cells and synergizes with PD-1 blockade to eradicate established tumors. Immunity. 2017;46(4):577–86. doi:10.1016/j.immuni.2017.03.013. 28410988 PMC5437702

[193] Mitsui J, Nishikawa H, Muraoka D, Wang L, Noguchi T, Sato E, Kondo S, Allison JP, Sakaguchi S, Old LJ, et al. Two distinct mechanisms of augmented antitumor activity by modulation of immunostimulatory/inhibitory signals. Clin Cancer Res. 2010;16(10):2781–91. doi:10.1158/1078-0432.CCR-09-3243. 20460483

[194] Ueha S, Yokochi S, Ishiwata Y, Ogiwara H, Chand K, Nakajima T, Hachiga K, Shichino S, Terashima Y, Toda E, et al. Robust antitumor effects of combined anti–CD4-Depleting antibody and anti–PD-1/PD-L1 immune checkpoint antibody treatment in mice. Cancer Immunol Res. 2015;3(6):631–640. doi:10.1158/2326-6066.CIR-14-0190. 25711759

[195] Geva R, Voskoboynik M, Dobrenkov K, Mayawala K, Gwo J, Wnek R, Chartash E, Long GV. First-in-human phase 1 study of MK-1248, an anti–glucocorticoid-induced tumor necrosis factor receptor agonist monoclonal antibody, as monotherapy or with pembrolizumab in patients with advanced solid tumors. Cancer. 2020;126(22):4926–4935. doi:10.1002/cncr.33133. 32809217

[196] Papadopoulos KP, Autio K, Golan T, Dobrenkov K, Chartash E, Chen Q, Wnek R, Long GV. Phase I Study of MK-4166, an Anti-human Glucocorticoid-Induced TNF Receptor Antibody, Alone or with Pembrolizumab in Advanced Solid Tumors. Clin Cancer Res. 2021;27(7):1904–1911. doi:10.1158/1078-0432.CCR-20-2886. 33355238 PMC9094061

[197] Davar D, Zappasodi R, Wang H, Naik GS, Sato T, Bauer T, Bajor D, Rixe O, Newman W, Qi J, et al. Phase IB study of GITR agonist antibody TRX518 singly and in combination with gemcitabine, pembrolizumab, or nivolumab in patients with advanced solid tumors. Clin Cancer Res. 2022;28(18):3990–4002. doi:10.1158/1078-0432.CCR-22-0339. 35499569 PMC9475244

[198] Hernandez-Guerrero T, Moreno V. GITR antibodies in cancer: not ready for prime time. Clin Cancer Res. 2022;28(18):3905–7. doi:10.1158/1078-0432.CCR-22-1489. 35834593

[199] Steele CW, Karim SA, Leach JDG, Bailey P, Upstill-Goddard R, Rishi L, Foth M, Bryson S, McDaid K, Wilson Z, et al. CXCR2 inhibition profoundly suppresses metastases and augments immunotherapy in pancreatic ductal adenocarcinoma. Cancer Cell. 2016;29(6):832–45. doi:10.1016/j.ccell.2016.04.014. 27265504 PMC4912354

[200] Greene S, Robbins Y, Mydlarz WK, Huynh AP, Schmitt NC, Friedman J, Horn LA, Palena C, Schlom J, Maeda DY, et al. Inhibition of MDSC trafficking with SX-682, a CXCR1/2 inhibitor, enhances NK-Cell immunotherapy in head and neck cancer models. Clin Cancer Res. 2020;26(6):1420–31. doi:10.1158/1078-0432.CCR-19-2625. 31848188 PMC7073293

[201] Horn LA, Riskin J, Hempel HA, Fousek K, Lind H, Hamilton DH, McCampbell KK, Maeda DY, Zebala JA, Su Z, et al. Simultaneous inhibition of CXCR1/2, TGF-β, and PD-L1 remodels the tumor and its microenvironment to drive antitumor immunity. J Immunother Cancer. 2020;8(1):e000326. doi:10.1136/jitc-2019-000326. 32188703 PMC7078948

[202] Nywening TM, Belt BA, Cullinan DR, Panni RZ, Han BJ, Sanford DE, Jacobs RC, Ye J, Patel AA, Gillanders WE, et al. Targeting both tumour-associated CXCR2+neutrophils and CCR2+macrophages disrupts myeloid recruitment and improves chemotherapeutic responses in pancreatic ductal adenocarcinoma. Gut. 2018;67(6):1112–1123. doi:10.1136/gutjnl-2017-313738. 29196437 PMC5969359

[203] Hornyák L, Dobos N, Koncz G, Karányi Z, Páll D, Szabó Z, Halmos G, Székvölgyi L. The role of indoleamine-2,3-dioxygenase in cancer development, diagnostics, and therapy. Front Immunol. 2018;9:151. doi:10.3389/fimmu.2018.00151. 29445380 PMC5797779

[204] Abd El-Fattah EE. Ido/kynurenine pathway in cancer: possible therapeutic approaches. J Transl Med. 2022;20(1):347. doi:10.1186/s12967-022-03554-w. 35918736 PMC9344609

[205] Yao Y, Liang H, Fang X, Zhang S, Xing Z, Shi L, Kuang C, Seliger B, Yang Q. What is the prospect of indoleamine 2,3-dioxygenase 1 inhibition in cancer? Extrapolation from the past. J Exp Clin Canc Res. 2021;40(1):60. doi:10.1186/s13046-021-01847-4. PMC7869231 33557876

[206] Long GV, Dummer R, Hamid O, Gajewski TF, Caglevic C, Dalle S, Arance A, Carlino MS, Grob J-J, Kim TM, et al. Epacadostat plus pembrolizumab versus placebo plus pembrolizumab in patients with unresectable or metastatic melanoma (ECHO-301/KEYNOTE-252): a phase 3, randomised, double-blind study. Lancet Oncol. 2019;20(8):1083–97. doi:10.1016/S1470-2045(19)30274-8. 31221619

[207] Robert DL, Leisha AE. Targeting adenosine for cancer immunotherapy. J Immunother Cancer. 2018;6(1):57. doi:10.1186/s40425-018-0360-8. 29914571 PMC6006764

[208] Thomas DA, Massagué J. TGF-β directly targets cytotoxic T cell functions during tumor evasion of immune surveillance. Cancer Cell. 2005;8(5):369–80. doi:10.1016/j.ccr.2005.10.012. 16286245

[209] Ahmadzadeh M, Rosenberg SA. TGF-beta 1 attenuates the acquisition and expression of effector function by tumor antigen-specific human memory CD8 T cells. J Immunol. 2005;174(9):5215–5223. doi:10.4049/jimmunol.174.9.5215. 15843517 PMC2562293

[210] Gunderson AJ, Yamazaki T, McCarty K, Fox N, Phillips M, Alice A, Blair T, Whiteford M, O’Brien D, Ahmad R, et al. TGFβ suppresses CD8+ T cell expression of CXCR3 and tumor trafficking. Nat Commun. 2020;11(1):1749. doi:10.1038/s41467-020-15404-8. 32273499 PMC7145847

[211] Batlle E, Massagué J. Transforming growth factor-β signaling in immunity and cancer. Immunity. 2019;50(4):924–40. doi:10.1016/j.immuni.2019.03.024. 30995507 PMC7507121

[212] Jaillon S, Ponzetta A, Di Mitri D, Santoni A, Bonecchi R, Mantovani A. Neutrophil diversity and plasticity in tumour progression and therapy. Nat Rev Cancer. 2020;20(9):485–503. doi:10.1038/s41568-020-0281-y. 32694624

[213] Lan Y, Zhang D, Xu C, Hance KW, Marelli B, Qi J, Yu H, Qin G, Sircar A, Hernández VM, et al. Enhanced preclinical antitumor activity of M7824, a bifunctional fusion protein simultaneously targeting PD-L1 and TGF-β. Sci Transl Med. 2018;10(424). doi:10.1126/scitranslmed.aan5488. 29343622

[214] Knudson KM, Hicks KC, Luo X, Chen JQ, Schlom J, Gameiro SR. M7824, a novel bifunctional anti-PD-L1/TGFβ trap fusion protein, promotes anti-tumor efficacy as monotherapy and in combination with vaccine. Oncoimmunology. 2018;7(5):e1426519. doi:10.1080/2162402X.2018.1426519. 29721396 PMC5927523

[215] Gameiro SR, Strauss J, Gulley JL, Schlom J. Preclinical and clinical studies of bintrafusp alfa, a novel bifunctional anti-PD-L1/TGFβRII agent: Current status. Exp Biol Med (Maywood). 2022;247(13):1124–34. doi:10.1177/15353702221089910. 35473390 PMC9335510

